# Binding, Conformational Transition and Dimerization of Amyloid-β Peptide on GM1-Containing Ternary Membrane: Insights from Molecular Dynamics Simulation

**DOI:** 10.1371/journal.pone.0071308

**Published:** 2013-08-09

**Authors:** Moutusi Manna, Chaitali Mukhopadhyay

**Affiliations:** 1 Department of Chemistry, University of Calcutta, Kolkata, India; 2 Department of Physics, Tampere University of Technology, Tampere, Finland; University of Akron, United States of America

## Abstract

Interactions of amyloid-β (Aβ) with neuronal membrane are associated with the progression of Alzheimer’s disease (AD). Ganglioside GM1 has been shown to promote the structural conversion of Aβ and increase the rate of peptide aggregation; but the exact nature of interaction driving theses processes remains to be explored. In this work, we have carried out atomistic-scale computer simulations (totaling 2.65 µs) to investigate the behavior of Aβ monomer and dimers in GM1-containing raft-like membrane. The oligosaccharide head-group of GM1 was observed to act as scaffold for Aβ-binding through sugar-specific interactions. Starting from the initial helical peptide conformation, a β-hairpin motif was formed at the C-terminus of the GM1-bound Aβ-monomer; that didn’t appear in absence of GM1 (both in fluid POPC and liquid-ordered cholesterol/POPC bilayers and also in aqueous medium) within the simulation time span. For Aβ-dimers, the β-structure was further enhanced by peptide-peptide interactions, which might influence the propensity of Aβ to aggregate into higher-ordered structures. The salt-bridges and inter-peptide hydrogen bonds were found to account for dimer stability. We observed spontaneous formation of intra-peptide D^23^-K^28^ salt-bridge and a turn at V^24^GSN^27^ region - long been accepted as characteristic structural-motifs for amyloid self-assembly. Altogether, our results provide atomistic details of Aβ-GM1 and Aβ-Aβ interactions and demonstrate their importance in the early-stages of GM1-mediated Aβ-oligomerisation on membrane surface.

## Introduction

Alzheimer’s disease (AD) is a progressive cognitive decline that pathologically characterized by the presence of senile plaques within the gray matter of brain. The neuritic plaques contain filamentous aggregates of amyloid-β (Aβ) peptides [1], proteolytically cleaved from transmembrane amyloid precursor protein. Aβ is 39–43 residue-long peptide and the C-terminal properties of Aβ critically determines its aggregation potential [2]. While Aβ_1–40_ composes approximately 90% of total secreted Aβ, the highly amyloidogenic 42-residue form Aβ_1–42_ is the principal component of the diffuse deposits [1]. Amyloid fibrils were long suspected as proximate disease agents, but recent compelling evidences suggest that small soluble oligomers of Aβ are the major neurotoxic components [3], [4]. A details structural characterization of early aggregation intermediates is thus essential for understanding the origin of AD and to develop therapeutic strategies. Although much is known about the molecular structure of Aβ-fibrils [5], [6], which universally exhibit a characteristic cross β-structure; the transient and disordered nature of low-ordered oligomers has made it difficult to pin down their structural properties and the dynamics of assembly pathway [7], [8]. The limited solubility and structural heterogeneity of the early Aβ-aggregates stand in the way of crystallization and limits experimental outcome, that average over different morphologies. Though Aβ has garnered much scientific attention, many fundamental aspects, such as how and where these soluble peptides are transformed into toxic oligomers and the cellular mechanism of toxicity - still remain elusive.

It has been proposed that the interaction of Aβ with neuronal membrane is integral to its toxicity and progression of AD [9], [10]. Over last few decades a great deal of efforts has been made to explore the behavior of Aβ within different lipid environments. Studies showed that Aβ could intercalate into the membrane and eventually cause severe membrane damage [11], [12]. Subsequent nonspecific membrane leakage or specific ionic transport through channels could perturb ion homeostasis and might be directly related to Aβ cytotoxicity [11]–[14]. While there have been other reports revealing the significant catalytic role of membrane in Aβ fibrillogenesis [10], [15]. Association of peptides on membrane surface increases the local peptide concentration and reduces their self-assembly dimension from three to two, thereby speed up the aggregation rate than would occur in solution [10], [15]. The compositional/lateral heterogeneity of biological membrane renders the study of Aβ-membrane interactions extremely complicated and gives rise to controversial mechanisms of Aβ induced toxicity in neuronal cells.

Lipid rafts, the cholesterol and sphingolipids enriched highly ordered membrane microdomains, are potential modulators of Aβ production, aggregation and toxicity [9]. Gangliosides are important components of lipid rafts and are known to play key role in the formation of amyloid fibrils by Aβ [9]. A recent study showed that the presence of ganglioside enhances both the early pore formation and the fibril-dependent membrane fragmentation process [11]. These sialic acid containing glycosphingolipids are abundant in plasma membrane of neurons and are involved in numerous neurobiological events [16]. Yanagisawa et at. had identified monosialoganglioside GM1-bound Aβ from AD brain and suggested the involvement of GM1 not only in aggregate formation but also in neurotoxic events [17]. Thereafter, a series of spectroscopic studies had revealed direct binding interactions between GM1 and Aβ [18]–[21]. GM1 was shown to facilitate the conformational transition of Aβ and accelerate amyloid fibril formation [20]–[23] and also these fibrils exhibited greater cytotoxicity than fibrils formed in solution [24]. Further, the formation of Aβ-GM1 complex is facilitated in raft-like environments that are enriched in cholesterol [10], [20]. However, despite rigorous experimental focus, the exact nature of GM1-Aβ interactions driving these processes, thus the underlying molecular-mechanism is not yet determined and the aggregation states of Aβ during these investigations are also unclear. Simulation is one of the most viable techniques to study complex biological systems and being widely used now a day [25]–[26]. A large number of simulation studies had investigated the structural properties of Aβ dimers/trimers in aqueous solution [8], [27]–[31], but there are very limited computational attempts to focus on how they behave in presence lipid membranes [32]–[34], especially within their native raft-like environment. Some recent computational studies had investigated the interactions of pre-formed Aβ protofilaments or preassembled fibril-like Aβ oligomers with one-component lipid bilayer [35], [36] or with self-assembled monolayers [37]; where these models were based on NMR-derived structures of Aβ-fibril with significant β-sheet content.

In the present work, we have performed MD simulation (totaling 2.65 µs) to investigate the effects of GM1 on the accumulation, conformational transition and subsequent dimerization of full length Aβ_1–42_ - which are the crucial early events during oligomerisation of Aβ on membrane surface. The goal of the present work is twofold. First we investigated the interactions of monomeric Aβ with GM1-containing raft-like membrane, composed of GM1/Chol/POPC. For comparison, two control simulations of Aβ monomer were performed in absence of GM1: (i) in liquid-ordered (L_o_) Chol/POPC (containing 25 mol% Chol) bilayer and (ii) in liquid-disordered (L_d_) POPC membrane. Another reference system of Aβ_1–42_ monomer in water was also simulated. We observed that the carbohydrate headgroup of GM1 acted as binding sites for Aβ and induced a β-hairpin structure at the C-terminus of the peptide. The α-helix to β-strand conformational transition is considered as the key step in the amyloidogenic oligomerisation process, as amyloid peptides must acquire β-sheet conformation to aggregate and polymerize [12]. To further examine whether the GM1-induced structure can form stable Aβ-oligomers, we had studied the structure and association pattern of three different dimeric arrangements of Aβ at the interface of GM1/Chol/POPC bilayer. Dimer formation is the first step in aggregation and has shown to be adversely toxic to neurons [4]. The results of the present simulations are compared with the available experimental data where possible.

## Methods

### System Setup

#### Lipid bilayers

Three model membranes with different lipid compositions (Table 1) were used in this study. These bilayers with varying fluidity (Table S1 and Text S1) can represent different regions of neuronal membrane and each was equilibrated for 150 ns. *(i) Single-component POPC bilayer*: The bilayer composed of unsaturated POPC lipids (*sn*-1 chain 16∶0 and *sn*-2 chain 18∶1) represented the fluid-phase membrane at room temperature (as phase transition temperature of POPC is 268 K) [25]. *(ii) Chol/POPC bilayer*: With addition of 25 mol% of Chol, the bilayer became more densely packed, with significant increase in bilayer rigidity and ordering [38]. *(iii) GM1/Chol/POPC bilayer*: Gangliosides are abundant in neuronal membrane and constitute 5–10% of lipids on the outer leaflet of cell membrane [39]. GM1 is an anionic glycosphingolipid containing a large penta-saccharide (four neutral sugar groups and one sialic acid) head group (Figure S1). Initially, coordinates for the ternary lipid-mixture: GM1/Chol/POPC were taken from a previously published work [40], where we had reported the formation of highly ordered GM1-Chol lateral assemblies. For the current study, the initial ternary system was enlarged (total 224 lipids) and equilibrated for further 150 ns (Table 1). In accordance with the experimental findings, here GM1 was present only in the extracellular leaflet, while we had maintained an equal concentration (25 mol%) of Chol in both layers. Our ternary bilayer closely mimicked the composition of lipid-rafts and properties of this bilayer (including very high lipid-tail order and bilayer mechanical strength, Table S1 and Text S1) were supportive of its liquid-ordered nature.

**Table 1:** The systems under MD simulation study.

	Number of each components							
Systems	Aβ	POPC	Chol	GM1	Water	Na^+^	Cl^−^	Simulation time (ns)
**POPC bilayer**	–	128	–	–	6014	16	16	150
**Chol/POPC bilayer**	–	116	38	–	7170	19	19	150
**GM1/Chol/POPC bilayer**	–	161	56	7	8790	30	23	150
**Aβ_42_/Water**	1	–	–	–	6929	22	19	250
**Aβ_42_/POPC**	1	128	–	–	6740	21	18	250
**Aβ_42_/Chol/POPC**	1	116	38	–	8757	26	23	250
**Aβ_42_/GM1/Chol/POPC**	1	161	56	7	9555	35	25	250
**Dimer1/GM1/Chol/POPC**	2	161	56	7	10402	40	27	400
**Dimer2/GM1/Chol/POPC**	2	161	56	7	10312	40	27	400
**Dimer3/GM1/Chol/POPC**	2	161	56	7	10704	41	28	400

#### Aβ-monomer model

Aβ peptide [D^1^AEFRHDSGY^10^EVHHQKLVFF^20^AEDVGSNK-GA^30^IIGLMVGGVV^40^IA] is comprised of hydrophilic N-terminus (residues: 1–28) and hydrophobic C-terminus (residues: 29–40). The starting coordinates of full-length Aβ_1–42_ were taken from PDB entry: 1Z0Q [41]. This aqueous solution structure of Aβ_1–42_ derived by solution NMR consisted of two helical domains (residues: 10–22 and 28–32) connected by a turn (residues: 25–26) (Figure S2a). To construct the Aβ-monomer-membrane systems, peptide was then placed parallel on the top of equilibrated bilayer surface, approximately 35 Å away from the average phosphate plane of the contact monolayer (Figure S2b). Aβ-membrane systems were energy minimized, followed by simulation with position restraint on the peptide heavy atom and then subjected to 250 ns long unrestraint production run (Table 1). In addition, Aβ-monomer was also simulated for 250 ns in aqueous solution (Table 1).

#### Initial dimer structure

The major problem associated with the structural characterization of amyloid aggregates is their heterogeneity. Amyloid fibrils can adopt distinct morphologies differing in shape, size and association pattern (parallel and anti-parallel β-sheet alignment) of Aβ units [5], [6]. Compared to fibrils/protofibrils, small oligomers of Aβ are less ordered and their structures are highly heterogeneous, including significant β-sheet, α-helix and unstructured sections [8], [27]–[29]. Despite ambiguities, emerging findings from earlier studies conjectured that – the hydrophobic C-terminus of Aβ peptide tends to adopt β-structure and form the central hydrophobic core, while the hydrophilic N-terminus is more solvent exposed [5], [6]. Though varieties of biophysical techniques provide valuable information about Aβ oligomers, their high-resolution structures are still not available. Previous MD simulations had revealed that the small oligomers, like dimers, trimers etc., are capable of adopting diverse variety of intra−/inter-molecular parallel and antiparallel stacking configurations, with possible lateral and frontal packing of peptide monomers [8], [27]–[32], [42].

Based on the previous findings, Aβ_42_-dimers with three different packing configurations were modeled in the present work (Figure S3). Our final structure of Aβ_42_ monomer bound to GM1/Chol/POPC bilayer served as starting configuration for Aβ_42_ units in dimers. Initially, each Aβ was separated by minimum center of mass distance of 1.6 nm. For first two cases, peptides were placed side-by-side, where their C-terminal β-hairpin segments were kept in adjacent positions, with antiparallel (dimer 1, Figure S3a) and parallel (dimer 2, Figure S3c) orientations. While in another dimer, peptides were stacked on the top of each other (dimer 3, Figure S3e). Dimers were then placed above (∼2.2 nm) the surface of GM1/Chol/POPC bilayer (Figure S3b,d,f), energy minimized to remove clashes within the system, equilibrated with position restraint on the peptide and finally followed by 400 ns unrestraint production run for each of the dimer-membrane systems (Table 1).

### Parameters and Simulation Protocols

All simulations were performed using GROMACS 3.3.1 package [43], [44] and the standard united-atom GROMOS87 force field [45]. The ffgmx force field was used to represent peptide in conjunction with Berger lipids [46]. This set of parameters was shown to perform well in many membrane simulations [38], [47]–[48]. The structure and topology of cholesterol molecule was taken from Höltje et al. [49], with the atomic charge modifications by Olsen et al. [50]. Parameters for GM1 were taken from Sega et al. [51]. The Ryckaert-Bellemans potential was used to model the torsional angles of lipid hydrocarbon tails [52]. The SPC model was used for water molecules [53]. To maintain the electro-neutrality of the system Na^+^ counter ions were added and additional NaCl was added to achieve the physiological salt concentration. All MD simulations were carried out under the isobaric–isothermal (NPT) ensemble with imposed 3D periodic boundary conditions. A time step of 2 fs was used for integrating the equation of motion. The Berendsen algorithm [54] was employed to keep temperature (at 300 K, using coupling constant τ_T_ = 0.1 ps) and pressure (semiisotropically at 1 bar, using coupling constant τ_P_ = 1.0 ps) constant. Lennard-Jones interactions were cutoff at a distance of 1.2 nm. For long-range electrostatics we had employed the particle mesh Ewald (PME) method [55] with a real-space cutoff of 1.2 nm. LINCS algorithm [56] was applied to constrain all bonds. VMD [57] and Pymol [58] were used for visualization.

## Results and Discussion

### Aβ-monomer Systems

#### Distribution of Aβ in bilayer environment

**Figure 1:**
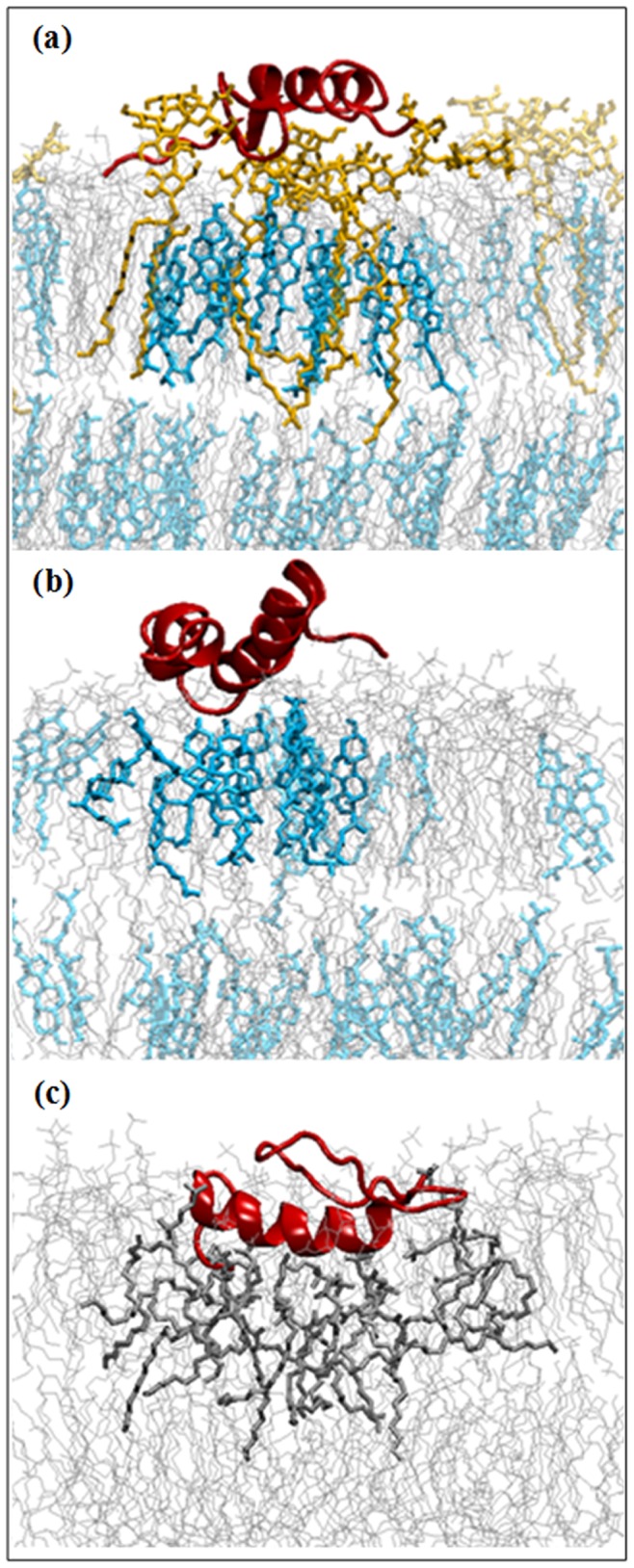
Final snapshots of membrane-bound Aβ_1–42_ monomer in different lipid bilayers. Aβ in (a) GM1/Chol/POPC, (b) Chol/POPC, and (c) POPC bilayers. Here, peptide was presented as red cartoon. Phospholipids, cholesterols and GM1 were shown in grey, cyan and orange, respectively. The lipids those were adjacent to the peptide made highlighted. Water molecules and ions were not shown for clarity.

**Figure 2:**
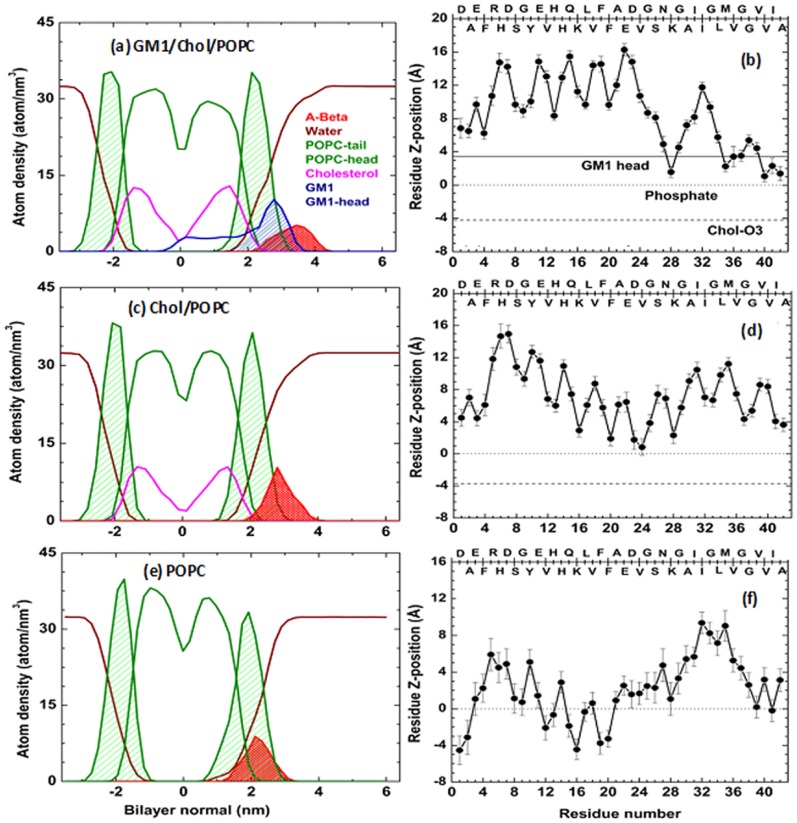
Location and orientation of Aβ-monomer at membrane-interfaces. (a,c,e) Atom density profiles (last 100 ns average) of peptide, lipids and water were plotted along Z-axis. For POPC (green) and GM1 (blue) the shaded area under the curve represented their head-group regions. (b,d,f) The average (last 100 ns) distance of the center of mass of peptide residues from bilayer interfaces. The horizontal lines represent the average planes of GM1 penta-saccharide head (solid line), Chol -OH oxygen (dashed line) and phosphorus atom of POPC (dotted line at Z = 0 position).

Figure 1 represents the final snapshots of membrane-bound Aβ in different bilayer environments. In GM1 containing membrane, Aβ resided at the oligosaccharide head group of GM1 (Figure 1a and Figure 2a). The voluminous carbohydrate moiety of GM1 prevented deeper insertion of the peptide to the bilayer core. The hydrophobic C-terminus of Aβ approached the membrane-water interface, while the rest of the peptide remained partly exposed to the aqueous environment (Figure 2b). Our results correlated well with ample experimental findings suggesting accumulation of Aβ in the carbohydrate region of ganglioside containing model membranes [19], [59]. In a previous NMR spectroscopic study, the observed change in NMR signals from the sugar moiety indicated perturbations in exactly the region where Aβ was located [59]. For our simulation of GM1-depleated Chol/POPC bilayer, the interfacial accumulation of Aβ was also observed (Figure 1b and Figure 2c); where the central part (Lys^16^–Lys^28^) of the peptide acted as glue to assist its surface association (Figure 2d). In contrary, the peptide got deeply inserted into the liquid-disordered POPC membrane (Figure 1c and Figure 2e). Many residues of the N-terminal segment of Aβ, including several charged/polar residues, penetrated well below (up to 5 Å) the average phosphate plane, with aromatic Phe^19^ and Phe^20^ residues favorably immersed into membrane hydrophobic interior (Figure 2f). Such orientation might attribute to Aβ-induced membrane perturbation [60]. Thus the positioning of Aβ_1–42_ in membranes was found to be governed by the lipid-composition and consequently its strength of interaction with membrane was observed to vary in the order: POPC>Chol/POPC>GM1/Chol/POPC (Figure S4).

#### Lipid-peptide interactions: Involvement of ganglioside-GM1

**Figure 3:**
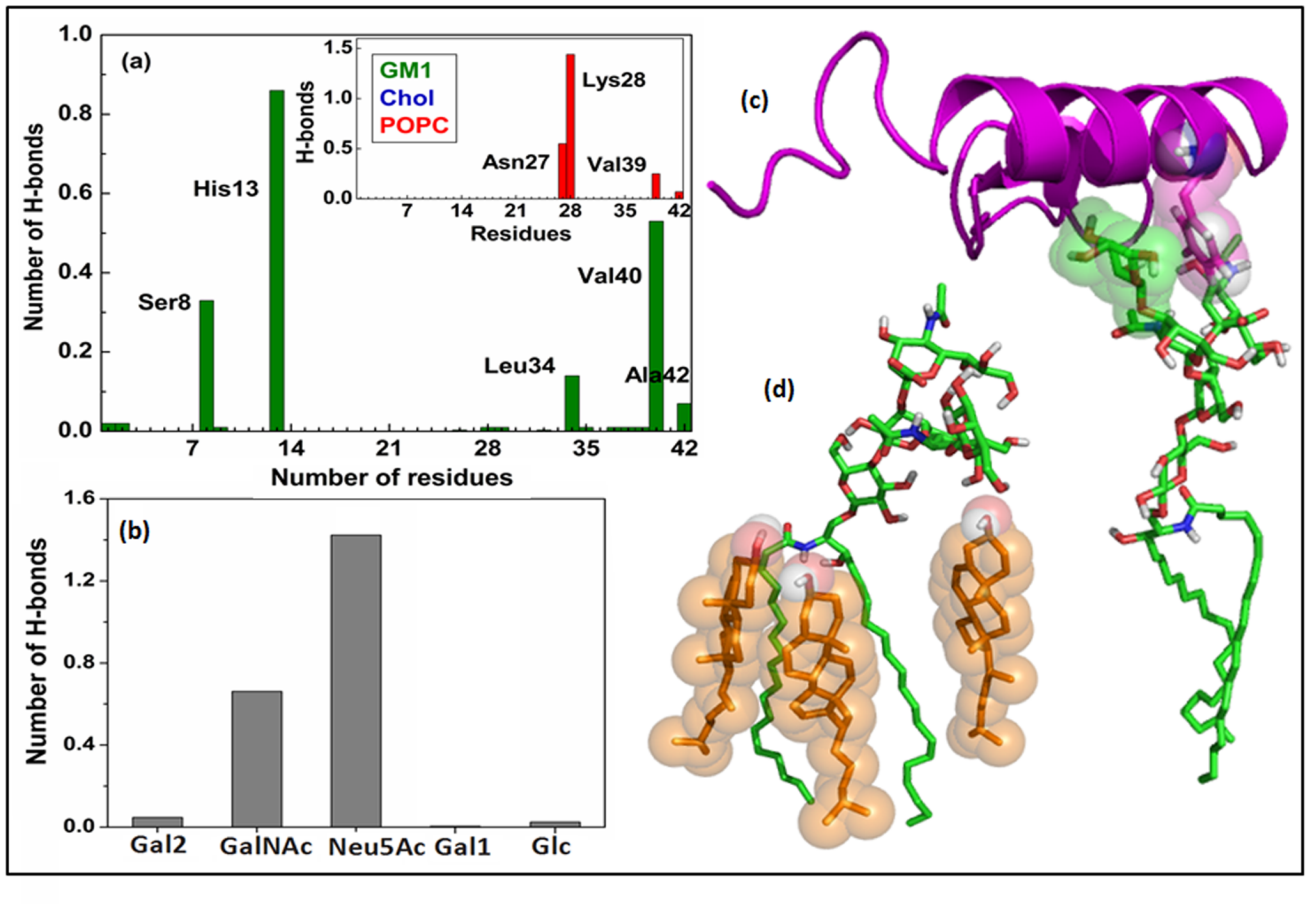
Interactions of Aβ with GM1-containing ternary bilayer. (a) Number of H-bonds formed by Aβ with GM1 (in green) and POPC (in red) in the ternary GM1/Chol/POPC bilayer. (b) Involvement of different sugar-residues in Aβ-GM1 interaction (the terminal galactose was termed as Gal2, whereas internal galactose was as Gal1). (c) Snapshot represents the CH-π stacking interaction between Phe^20^ and the pyranosyl ring of terminal galactose. Here Aβ was shown in purple cartoon and GM1 as green sticks. (d) Figure showed how cholesterol (in orange) constrained GM1 head-group conformation.

To identify specific lipid-Aβ interactions, the hydrogen bond (H-bond) participation of the peptide with the surrounding lipid molecules (Figure 3a and Figure S5) were calculated. An H-bond was defined by an acceptor-hydrogen distance less than 2.8 Å and a donor-hydrogen-acceptor angle greater than 120° [38]. In GM1-containing membranes both GM1 and POPC formed direct H-bonds with Aβ (Figure 3a). The basic His^13^, polar Ser^8^ and C-terminal hydrophobic residues, like, Leu^34^, Val^40^, Ala^42^ of the peptide were H-bonded with GM1 sugar groups (Figure 3a and Table S2). While residues like, Asn^27^, Lys^28^, Val^39^, those penetrated deeper into the membrane, formed H-bonds with polar head-groups of POPC (Figure 3a). Recently, His^13^/His^14^ has been identified as key residues for binding Aβ to GM1 [61], [62]. The penta-saccharide headgroup of GM1 (Figure S1) can provide numerous sites for the hydrogen bonding to the peptide. Further scrutiny for the involvement of different sugar moieties showed that Neu5Ac (i.e. sialic acid) has the major contribution for binding Aβ to the membrane, while GalNAc also significantly participate in H-bonding (Figure 3b). Our findings were consistent with previous experiments that reported an important role of sialic acid in ganglioside-specificity of Aβ. [20]. In addition, we were also able to capture CH-π stacking interaction between Phe^20^ and the terminal galactose ring (Figure 3c). The pyranosyl ring of galactose has two chemically distinct faces: one apolar face with hydrocarbyl (CH) groups and other polar face with –OH groups. The apolar side of galactose acted as complimentary surface for stacking interactions with aromatic residue (Figure 3c). The current findings were in line with earlier reports showing that the glycosphingolipid-binding domain should contain basic and aromatic residues to mediate specific glycosphingolipid-protein interactions [61].

In GM1-containing membrane, no direct interaction between Chol and Aβ was observed (Figure 3a). The hydroxyl (-OH) group of Chol was found to form several polar interactions with GM1 oligosaccharide head group as well as -NH and –OH of sphingosine and restricts the conformation of glycan moiety of GM1 to be significantly tilted towards membrane plane (Figure 3d). Fantini et al. had showed that such Chol-induced glycolipid conformation is crucial for optimum recognition of Aβ [63]. In absence of GM1 (in Chol/POPC bilayer), Chol directly interacted with Aβ and formed hydrogen bonds mainly with the basic Lys^16^ and Lys^28^ residues of peptide (Figure S5). The charged residues may acted as hook to initiate the early binding of Aβ to the bilayer and found to be crucial for Aβ-membrane interaction [64]–[66].

#### Effects on membrane morphology

**Figure 4:**
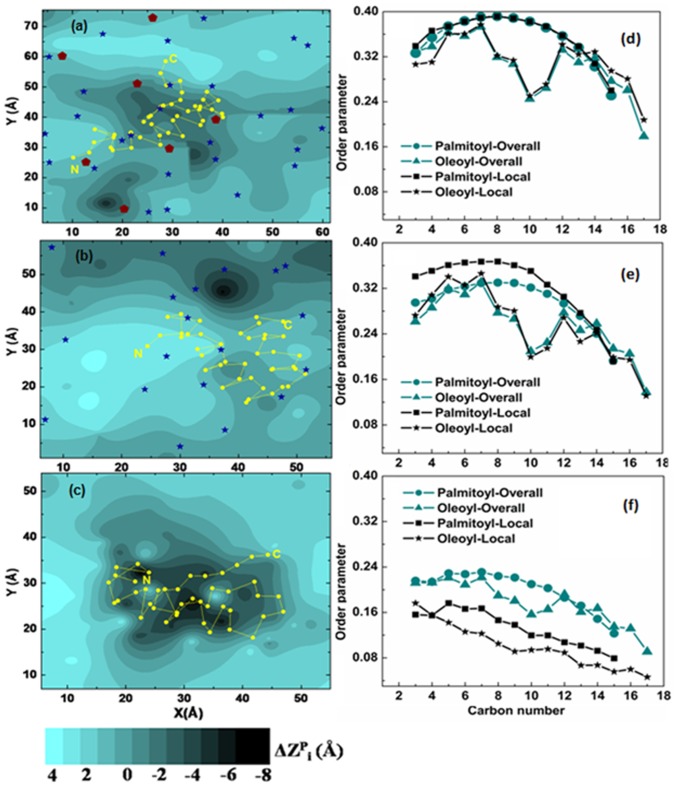
Effects of Aβ binding on bilayer properties. (a–c) The surface distribution of the change in bilayer thickness (defined in terms of Z coordinates of phosphorus atoms of individual POPC lipids as a function of their average in-plane positions) around Aβ in (a) GM1/Chol/POPC (b) Chol/POPC and (c) POPC bilayers. The average (X-Y) positions of peptide C_α_ atoms, Chol –OH oxygen and oxygen atom of glycosidic linkage of GM1 connecting ceramide tail with sugar head group, were shown in yellow (circle), blue (stars) and maroon (pentagons), respectively. (d-f) Order parameters of POPC lipid tails in (d) GM1/Chol/POPC (e) Chol/POPC and (f) POPC bilayers (last 100 ns average). To assess the effect of peptide, proximal lipids [i.e. local lipids, which with a lipid non-hydrogen atom within 10 Å of a peptide non-hydrogen atom] were considered separately.

Binding of Aβ exerted reciprocal effects on membrane biophysical properties, which were depicted by (i) the in-plane distribution of ΔZ_i_^P^ = Z_i_^P^−<Z^P^>, where Z_i_^P^ was Z-coordinates of the phosphorus atom of i^th^ POPC molecule in the contact monolayer and <Z^P^> was the average Z-value of this surface (Figure 4a–c) [47] and (ii) the molecular order parameter, S_mol_, of POPC acyl tails (Figure 4d–f). In POPC bilayer the deeper penetration of peptide strongly perturbed the local bilayer structure (Figure 4c,f). Lipids adjacent to the peptide become highly disordered, as depicted from the drastic drop in the order parameter values (Figure 4f). Simultaneously we observed formation of grooves or dents over a broad region underneath the peptide, with some portion of the bilayer even depressed up to 6–8 Å (Figure 4c). Such membrane-destabilizing effects of Aβ often can damage bilayer integrity and are known to be precursor to the formation of pore structures and ion channels. With the incorporation of 25 mol % Chol, the bilayer became more ordered and densely packed (Table S1); that prevented the deeper insertion of peptide (Figure 1b and Figure 2c) and protected membrane from Aβ-induced perturbation (Figure 4b,e). Since in Chol/POPC bilayer, Aβ was found to bind at the Chol-rich region (Figure 1b), two opposing effects were operating at the peptide contact area: (1) Aβ-induced membrane destabilization and (2) Chol-induced bilayer ordering; here the latter predominated over the other. Moreover the greater increase in the ordering of palmitoyl tail was probably due to the better packing of Chol around the saturated tail rather than the unsaturated one (Figure 4e). With addition of GM1 the rigidity of the ternary GM1/Chol/POPC bilayer was enhanced further (Table S1, Figure 4d). Here Aβ was found to bind at the interface of four GM1 molecules (Figure 4a) and remained partially solvent exposed. Such distribution of Aβ on membrane surface might increase the possibility of peptide-peptide interactions leading to aggregation. The interior of GM1-containing membrane remained almost unperturbed on Aβ invasion (Figure 4a,d). Thus, lipid-composition/bilayer fluidity can modulate the mode of Aβ-membrane interactions, responsible for the diverse mechanisms of Aβ-induced toxicity in neural cells.

#### Peptide conformation

**Figure 5:**
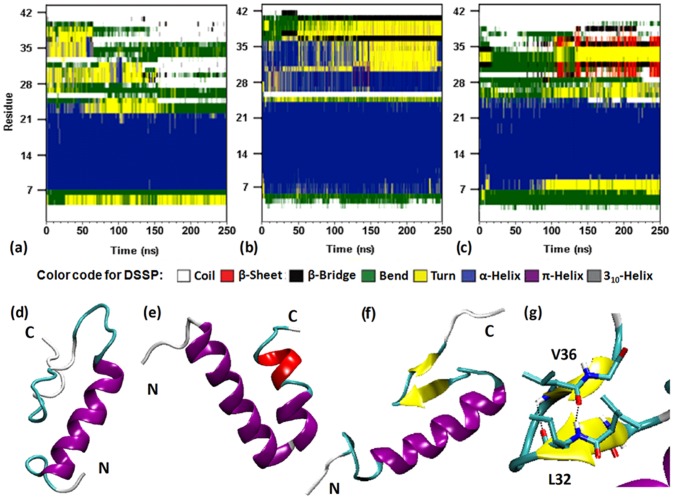
Membrane-induced structural conversion of Aβ-monomer. The secondary structure profiles of Aβ in (a) POPC, (b) Chol/POPC, and (c) GM1/Chol/POPC membranes. The final snapshots of Aβ on (d) POPC, (e) Chol/POPC, and (f) GM1/Chol/POPC bilayer surfaces. (g) H-bonds stabilize the β-hairpin structure. The default color code for DSSP plots (a-c)- random coil: white, β-sheet: red, β-bridge: black, bend: green, turn: yellow, α-helix: blue, π-helix: purple, 3_10_-helix: gray. In snapshots (d-g), the secondary structures of Aβ were colored based on the default representation in VMD (α-helix: purple, 3_10_-helix: blue, π-helix: red, extended-β: yellow, bridge-β: tan, turn: cyan, coil: white).

The structural transition of Aβ is a central step in amyloidogenic oligomerisation process. To investigate the impact of lipid-composition on peptide secondary structural content, we have plotted the secondary structure profile of Aβ at various bilayer interfaces (Figure 5a,b,c), calculated on the basis of DSSP (defined secondary structure of protein) program [67]. In GM1-containing membrane, the N-terminal part almost retained its overall helicity (Figure 5c). While we observed the formation of an anti-parallel β-sheet near peptide C-terminus (Figure 5c,f) that remained immersed into the membrane-water interface, surrounded by GM1 sugar moieties (Figure S6). The β-sheet segment was formed by two β-strands composed of residues Ala^30^-Ile^31^-Ile^32^ and residues Val^36^-Gly^37^, linked by a turn [residues: Gly^33^-Leu^34^-Met^35^] (Figure 5c,f). The inter-strand H-bonds (backbone): between –NH of Val^36^ with –C = O of Ile^32^ and also between –C = O of Val^36^ with –NH of Ile^32^ (Figure 5g), stabilized the β-hairpin-like structure and allowed it to evolve over last 150 ns of simulation. In contrary to the GM1-containing membrane, in POPC (Figure 5a,d) and Chol/POPC bilayers (Figure 5b,e) we did not observe appearance of any β-strand component even after 250 ns of simulations. No significant β-structure was also formed in aqueous solution (Figure S7). It has been reported earlier that GM1 amplifies the α-helix to β-sheet conformational transition of Aβ and facilitates the formation of toxic β-sheet-rich fibrils [20], [22]. Such conformational change of Aβ was not detected in presence of ganglioside-free vesicles composed of various phospholipids and sphingomyelin (as characterized by CD-spectroscopy) [21], [68]. In presence of anionic lipid, a recent study had reported formation of small β-hairpin in Aβ [66]. The helix/β-sheet mixed conformations of Aβ was previously predicted as possible intermediates for Aβ-oligomerisation [69]. However, the experimental studies were unable to distinguish the monomeric and different oligomeric states of Aβ. Owing to the high aggregation propensity of Aβ, small oligomers are hard to detect experimentally. Thus, to further investigate the role of GM1 in the early-stages of Aβ-aggregation, it was imperative to investigate whether the GM1-induced structure of monomeric Aβ_42_ can trigger ordered oligomer formation with increased inter-peptide interactions.

### Aβ_42_-dimers on GM1-containing Ternary Membrane

According to the nucleated polymerization model [70], the assembly of monomeric Aβ into oligomeric structure is crucial for amyloid-fibril formation. Here we have investigated the structure, association pattern and stabilizing forces of three different dimers on the surface of GM1-containing membrane. Our results showed that all dimers were stable over 400 ns simulation time scale and exhibited no tendency to get dissociated. All of these structures were well equilibrated, as can be seen by the leveling off of RMSD after 50 ns (Figure S8). These structures were stabilized by inter-peptide contact and hydrogen-bonding interactions and the initial separation distance between two Aβ was dropped down to <1.3 nm (Table S3). Energetically, Dimer1 exhibited strongest peptide-peptide interaction and was the most stable among all (Figure S9, Table S3 and Text S2). The results of Dimer1 were presented in the main text, while that of other two dimers were given in the supporting information. Moreover all three dimers were favorably accumulated on membrane surface (Figure S10, Table S4 and Text S3).

#### Secondary structure of Aβ_42_ dimers

**Figure 6:**
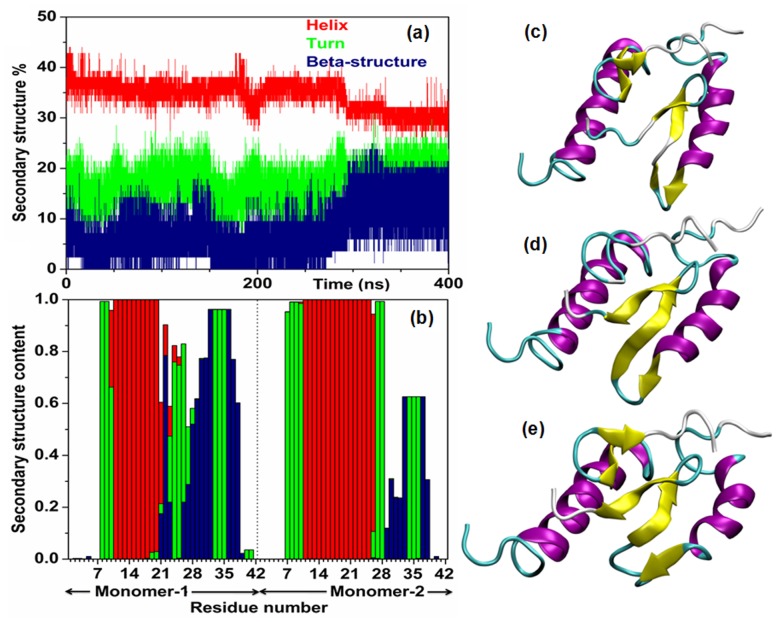
Secondary structures of Aβ-dimer. (a) Time profile of secondary structural contents and (b) average (last 125 ns) secondary structural content per residues of Dimer1 (calculated from DSSP); plotted with helix (sum of α-, 3_10_- and π- helices) in red, turn in green and β-structure (sum of extended β-strands and isolated β-bridges) in blue. Snapshots of Dimer1 at (c) 200 ns, (d) 275 ns and (e) 400 ns. In snapshots, the secondary structures of Aβ were colored based on the default representation in VMD (α-helix: purple, 3_10_-helix: blue, π-helix: red, extended-β: yellow, bridge-β: tan, turn: cyan, coil: white).

**Figure 7:**
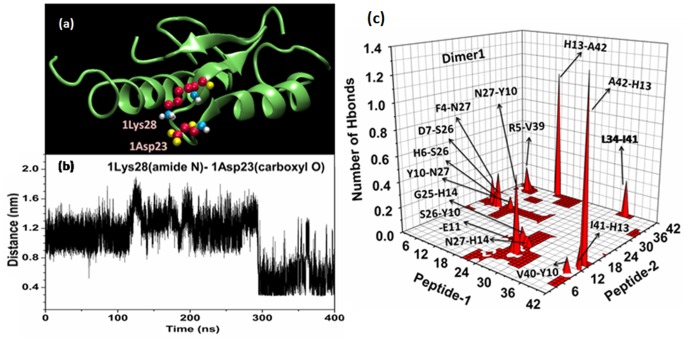
Factors accounting for dimer stability. (a) The snapshot showed the presence of intra-molecular Lys^28^-Asp^23^ slat-bridge in monomer-1 of Dimer1. Here the corresponding Lys^28^ and Asp^23^ were colored according to atom type: C in red, O in yellow, N in blue and H in white. (b) The time dependence of minimum distance between amide N of Lys^28^ and carboxyl O of Asp^23^ in monomer-1 of Dimer1, as highlighted in Figure 7a. (c) 3D-plot showing the inter-peptide hydrogen-bonding interactions within Dimer1 (last 200 ns average). The notation used here for leveling H-bond between a pair of residues was XA-YB, where X was the amino acid residue of peptide-1 with its corresponding residue number A and Y was the residue of peptide-2 with its corresponding residue number B.

To characterize the structural changes associated with the first step of assembly from monomeric to dimeric states, the secondary structure contents of dimers were plotted as function of time (Figure 6a, Figure S11a,c). We observed that significant amount β-structure persists for all dimers, along with turn and helical contents (Figure 6 and Figure S11). As depicted in Figure 6, dimerization appears to involve an increase in β-structure. For Dimer1, the β-content reached ∼20% during last 100 ns simulation (Figure 6a) and was in nice agreement with previous estimates: ∼15–30% from Circular Dichroism (CD) and NMR spectroscopy [18], [71] and ∼15–26% from simulations [8], [27]. All dimers were found to contain β-sheets at C-terminus formed between different sets of residues and with different interlocking patterns (Figure 6 and Figure S11). The average secondary structure probabilities of each residues of dimer1 were plotted in Figure 6b; where we observed the formation of well-reserved β-strands connected by short loops at peptides’ C-terminus. Interestingly, the occurrence of turn at V^24^-N^27^ region (Figure 6b) was in nice agreement with solid-state NMR fibril studies and complimentary MD simulations [6], [72]. The D^23^-K^28^ salt-bridge stabilizes the turn conformation (Figure 7a) (discussed in details later). The structure with intact D^23^-K^28^ salt-bridge and conserved V^24^GSN^27^ turn has long been identified as aggregation-prone structure [72], [73]. As depicted in Figure 6, β-strands in dimer were up to 5–7 residues long, hence were more extended than isolated monomers. Some β-strands were tending to be curved (Figure 6), perhaps lacking enough interactions to stabilize the flat extended sheets observed in mature fibrils. In a recent study the formation of a short segment of parallel intermolecular β-sheet was reported during assembly of three Aβ peptides in a mixed bilayer [33]. The small oligomers of Aβ do not have β-sheet structure characteristics of fibril, rather are composed of several shorter and loosely aggregated stands, which might polymerize in later stages during fibrillization [5].

#### Stabilizing forces

Characterization of driving forces for amyloid aggregation, in terms of key intra- and inter-peptide interactions, is an outstanding problem. According to the principal of amyloid self-assembly: maximizing hydrophobic and favorable electrostatic interactions enhanced fibril stability [73]. Salt bridges can play crucial role in oligomerisation and in stabilizing fibril-like structures. In the present work, we had calculated the formation time percentage of possible salt-bridges during the dimer trajectories and had listed in Table S5. A salt-bridge was considered when the distance between an amide N and a carboxyl O is less than 4.5 Å [38]. Interestingly, we observed spontaneous formation of intra-molecular D^23^-K^28^ salt-bridge in Dimer1 (Figure 7a,b) over 290 ns to 400 ns simulation time span. The D^23^-K^28^ salt-bridge was also found to be present in NMR-derived structures of Aβ-fibrils [5] and its importance has been emphasized in recent studies [73], [74]. In addition, we found that intra-molecular E^22^-K^28^ salt-bridge (in Dimer1) as well as inter-molecular salt-bridges between: K^28^-E^11^ and K^28^-E^3^ in Dimer1; R^5^-E^3^ and R^5^-D^7^ in Dimer2 and R^5^-D^1^, K^16^-D^7^ and K^28^-E^22^ in Dimer3 were also populated (Table S5). Both intra- and inter-peptide charge-pairs have significant contributions in dimer stability.

The interface of dimer is characterized by electrostatic interactions. Figure 7c, Figure S12 and Figure S13 depict the inter-peptide hydrogen bonds, which helped two Aβ-monomers to stick together within the dimers. For Dimer1, the predominant H-bonds formed between: H^13^(1)-A^42^(2) (numbers in brackets indicate peptide number within dimers, i.e., 1 for peptide-1 and 2 for peptide-2), A^42^(1)-H^13^(2), N^27^(1)-Y^10^(2) and L^34^(1)-I^41^(2) (Figure 7c). The charged/polar residues of dimers, like, D^1,7^, E^3,11,22^, H^6,13,14^, Y^10^, S^26^, N^27^ and K^28^, exhibited high H-bonding propensity (Figure 7c, Figure S12 and Figure S13) and their side-chains were mainly involved in the bonding network (Table S6, Table S7 and Table S8). Additionally, we observed important participation of many hydrophobic residues (like, A^2,21,30,42^, I^31,41^, L^34^, G^38^ and V^39^) in H-bond (main-chain) formation (Figure 7c, Figure S12, Figure S13 and Table S6–S8). Among them the C-terminus residues I^41^ and A^42^ are well known for their crucial role in the stability and toxicity of Aβ-fibrils [2]. Detailed knowledge about specific interactions may facilitate designing of possible inhibitors of Aβ-aggregation and developing therapeutic strategies against AD.

The hydrophobic, hydrophilic and total solvent-accessible surface area (SASA) for monomer and all dimers were also measured (Table S9). The significant drop in SASA values (by 16.85 nm^2^ for Dimer1, 14.93 nm^2^ for Dimer2 and 15.39 nm^2^ for Dimer3) upon monomer to dimer conversion indicated favorable peptide-association. Together, all these factors conferred stability to the dimers and might be important for protofibril/fibril formation in later stages.

### Conclusions

The behavior of Aβ-peptide within the cell membrane is integral to the manifestation of Alzheimer’s disease. Membrane composition defines the Aβ-lipid interactions and has significant implications in the context of age- and disease-related evolution of brain lipid expression and Aβ-deposition in AD [61]. We found that Aβ buried itself into the liquid-disordered POPC membrane and significantly perturbed membrane structure as a consequence of strong peptide-lipid interactions. Inclusion of cholesterol enhanced bilayer rigidity and protected membrane from Aβ-induced disruption. With addition of GM1, bilayer became more densely packed. The oligosaccharide head group of GM1 acted as privileged sites for Aβ binding- aided by hydrogen-bonding and CH-π stacking interactions with nearby sugar moieties. Such distribution of Aβ at the membrane-water interface may promote further peptide-peptide interactions characteristic of aggregation. Notably, the GM1-containing membrane exhibited a significant control over the process of β-strand formation in Aβ. For GM1-bound Aβ, we observed spontaneous formation of β-hairpin at the C-termini (residues: Ala^30^-Gly^37^) of the peptide. As the toxic Aβ-oligomers and mature fibrils are rich in β-strand content, the formation of β-structure has long been considered to be central to the aggregation cascade.

For our simulations of Aβ-dimers on GM1-containing membrane, the overall β-strand content was found to enhance considerably upon Aβ-Aβ interaction. Amyloid formation is a slow process (needs several hours to days) [11], [24] and still far beyond the reach of current atomistic simulation studies. However, it is worth noting that we had started with a helical peptide structure (PDB entry: 1Z0Q) without any β-strand content, while ended up with dimers having considerable β-structure, e.g., ∼20% β-content in Dimer1- in nice agreement with previous experiments. Our results showed that dimerization was favored by several inter- and intra-peptide salt-bridges and peptide-peptide hydrogen bonds. These forces can influence the propensity of Aβ to aggregate into higher-ordered structure. In Dimer1, we observed spontaneous formation of key structural elements, like, intra-peptide D^23^-K^28^ salt-bridge and turn at V^24^GSN^27^ region, which nucleate and stabilize the β-hairpin like structure and might act as template for larger oligomer growth. Altogether, the present work provides molecular level insight into the effects of ganglioside-GM1 on Aβ-membrane association, peptide’s conformational transition and aggregation- the three most vital early stages of membrane-induced Aβ-fibrillogenesis.

## Supporting Information

Figure S1 **Structure of ganglioside GM1.** GM1 [Gal β (1–3) GalNAc β (1–4) [Neu5Ac α (2–3)] Gal β (1–4) Glc β1-Ceramide] contains an oligosaccharide head group composed of: glucose (Glc), internal galactose (termed as Gal1), *N*-acetylneuraminic acid (Neu5Ac) or sialic acid, *N*-acetylgalactosamine (GalNAc), and terminal galactose (termed as Gal2).
(TIF)

Figure S2 **Description of initial structures for Aβ-monomer systems.** (a) Starting structure of Aβ_1–42_ (PDB entry: 1Z0Q) and (b) its initial position with respect to bilayer surface. The dashed line represented the average phosphate plane of the upper bilayer leaflet.
(TIF)

Figure S3 **Initial structures of three different dimers.** In (a) Dimer1 and (c) Dimer2: peptides were placed side-by-side, where their C-terminal β-hairpin segments were in adjacent positions, with antiparallel and parallel orientations, respectively. In (e) Dimer3: peptides were stacked on the top of each other. The right column showed the initial snapshots of dimers: (b) Dimer1, (d) Dimer2 and (f) Dimer3, placed on the top of GM1/Chol/POPC bilayer. The image rendering was done with VMD. Here peptides were shown in cartoon and their secondary structures were colored based on the default representation in VMD (α-helix: purple, 3_10_-helix: blue, π-helix: red, extended-β: yellow, bridge-β: tan, turn: cyan, coil: white). Phospholipids were shown as thin gray lines, cholesterols as green sticks and GM1 as orange van der Waals spheres.
(TIF)

Figure S4 **Lipid-peptide interaction energies in three different Aβ-monomer-membrane systems.** The average values of last 100 ns data were presented here.
(TIF)

Figure S5 **Number of Aβ-monomer-lipid H-bonds.** H-bonds in (a) Chol/POPC and (b) POPC bilayers.
(TIF)

Figure S6 **Aβ-monomer bound at the interface of four GM1 molecules.**
(TIF)

Figure S7 **Secondary structure of Aβ-monomer in aqueous solution.** (a) The secondary structure profile of Aβ in aqueous solution based on DSSP calculation. (b) The final snapshot of Aβ at 250 ns, prepared by VMD. The default color code of DSSP plot (a): random coil: white, β-sheet: red, β-bridge: black, bend: green, turn: yellow, α-helix: blue, π-helix: purple, 3_10_-helix: gray. In snapshot (b) the secondary structures were colored based on the default representation in VMD (α-helix: purple, 3_10_-helix: blue, π-helix: red, extended-β: yellow, bridge-β: tan, turn: cyan, coil: white).
(TIF)

Figure S8 **Root mean square deviations (RMSD) plotted against time for all three dimers.**
(TIF)

Figure S9 **Aβ-Aβ interaction energies in dimers (last 200 ns average).**
(TIF)

Figure S10 **Preferential location of Aβ-dimers on GM1-contating membrane surface.** Time dependence of the distance between the center of mass of Aβ-dimers and the average plane of phosphorus atoms in the contact monolayer.
(TIF)

Figure S11 **Secondary structures of Aβ-dimers.** Time profile of secondary structural contents of (a) Dimer2 and (c) Dimer3 and their snapshots: (b) Dimer2 and (d) Dimer3 near the end of simulation. In snapshots the secondary structures of Aβ were colored based on the default representation in VMD (α-helix: purple, 3_10_-helix: blue, π-helix: red, extended-β: yellow, bridge-β: tan, turn: cyan, coil: white).
(TIF)

Figure S12 **Inter-peptide H-bonds in Dimer2.** 3D-plot showing the inter-peptide hydrogen-bonding interactions within Dimer2 (last 200 ns average). The notation used here for leveling H-bond between a pair of residues was XA-YB, where X was the amino acid residue of peptide-1 with its corresponding residue number A and Y was the residue of peptide-2 with its corresponding residue number B.
(TIF)

Figure S13 **Inter-peptide H-bonds in Dimer3.** 3D-plot showing the inter-peptide hydrogen-bonding interactions within Dimer3 (last 200 ns average). The notation used was same as Figure S12.
(TIF)

Table S1 **Properties of equilibrated bilayers.**
(DOC)

Table S2 **Details of crucial Aβ-monomer-GM1 hydrogen bonding interactions.**
(DOC)

Table S3 **Properties of dimers (averaged over last 200 ns trajectory) on GM1-containing membrane.**
(DOC)

Table S4 **Interaction of dimers with membrane surface (averaged over last 200 ns trajectory).**
(DOC)

Table S5 **The formation time percentage (last 200 ns average) of intra- and inter-molecular salt-bridges in Aβ-dimers.** Listed were those, which had more than 10% existence.
(DOC)

Table S6 **Details of inter-peptide hydrogen-bonding interactions within Dimer1.** Listed were those which have H-bond ≥0.1.
(DOC)

Table S7 **Details of inter-peptide hydrogen-bonding interactions within Dimer2.** Listed were those which have H-bond ≥0.1.
(DOC)

Table S8 **Details of inter-peptide hydrogen-bonding interactions within Dimer3.** Listed were those which have H-bond ≥0.1.
(DOC)

Table S9 **Solvent-accessible surface area (SASA) for monomer (M) and dimers (D).**
(DOC)

Text S1 **Description of additional data for Table S1.**
(DOC)

Text S2 **Description of additional data for Table S3 and Figure S9.**
(DOC)

Text S3 **Description of additional data for Table S4 and Figure S10.**
(DOC)
