# Supplementary figures and images for: Binding, Conformational Transition and Dimerization of Amyloid-β Peptide on GM1-Containing Ternary Membrane: Insights from Molecular Dynamics Simulation
Source: PLoS One. 2013 Aug 9;8(8):e71308. doi: 10.1371/journal.pone.0071308 (PMC3739818; doi:10.1371/journal.pone.0071308)

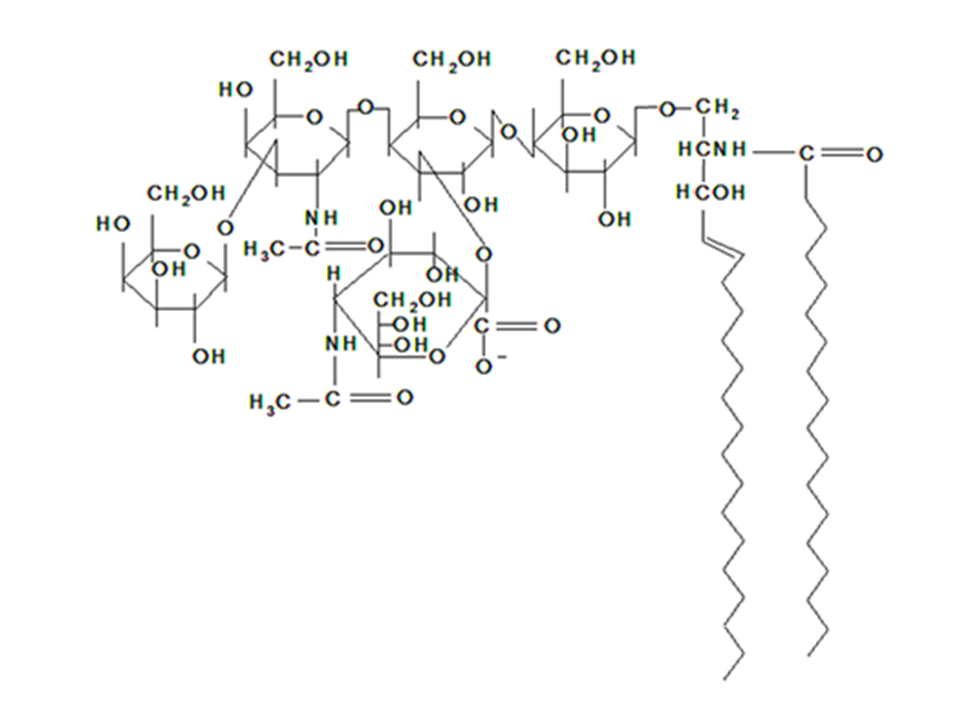

Supplement: Figure S1 — Structure of ganglioside GM1. GM1 [Gal β (1–3) GalNAc β (1–4) [Neu5Ac α (2–3)] Gal β (1–4) Glc β1-Ceramide] contains an oligosaccharide head group composed of: glucose (Glc), internal galactose (termed as Gal1), N-acetylneuraminic acid (Neu5Ac) or sialic acid, N-acetylgalactosamine (GalNAc), and terminal galactose (termed as Gal2). (TIF) [file pone.0071308.s001.tif]

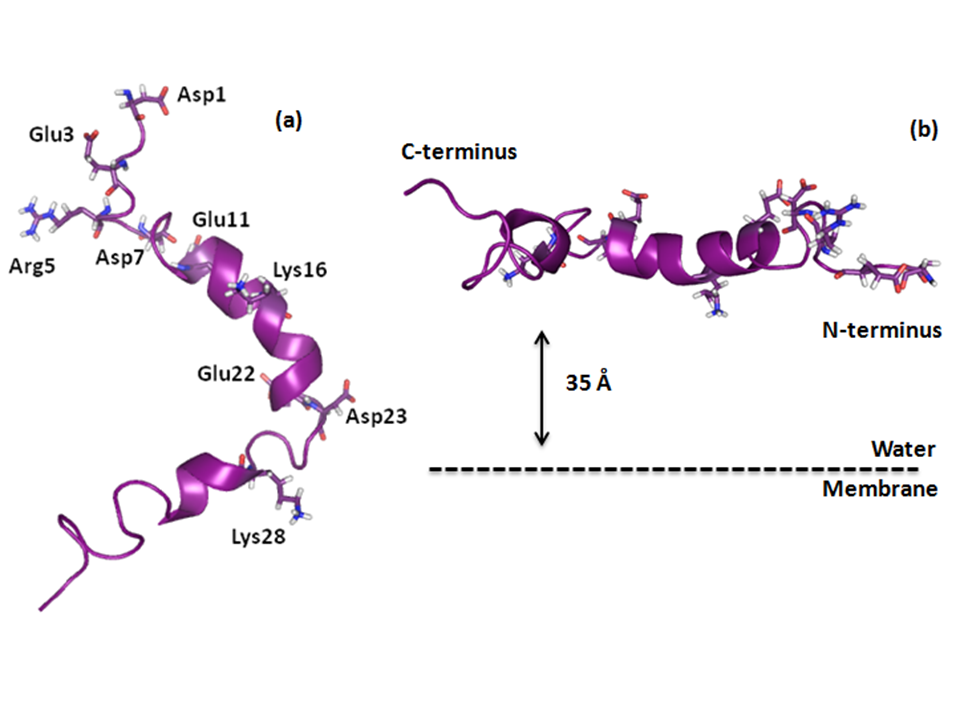

Supplement: Figure S2 — Description of initial structures for Aβ-monomer systems. (a) Starting structure of Aβ1–42 (PDB entry: 1Z0Q) and (b) its initial position with respect to bilayer surface. The dashed line represented the average phosphate plane of the upper bilayer leaflet. (TIF) [file pone.0071308.s002.tif]

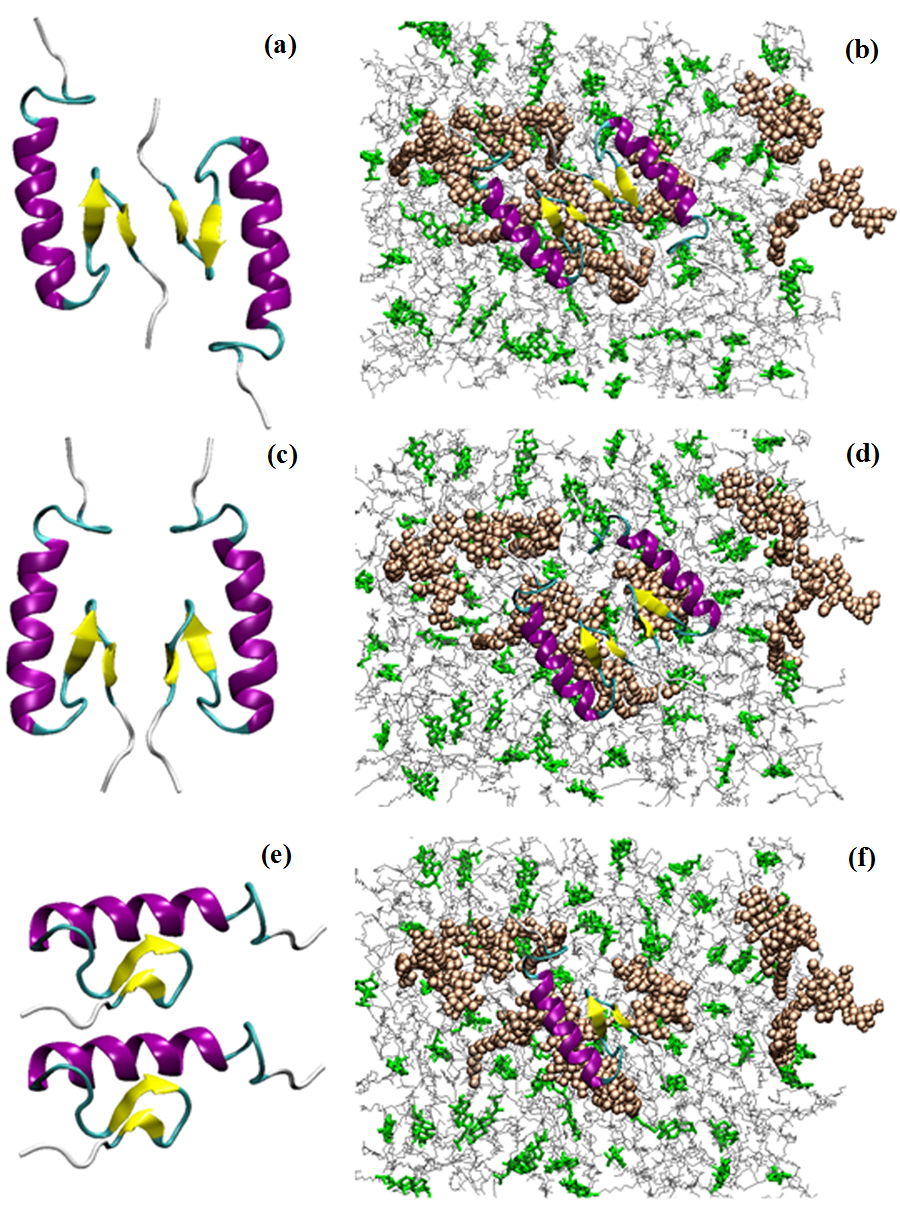

Supplement: Figure S3 — Initial structures of three different dimers. In (a) Dimer1 and (c) Dimer2: peptides were placed side-by-side, where their C-terminal β-hairpin segments were in adjacent positions, with antiparallel and parallel orientations, respectively. In (e) Dimer3: peptides were stacked on the top of each other. The right column showed the initial snapshots of dimers: (b) Dimer1, (d) Dimer2 and (f) Dimer3, placed on the top of GM1/Chol/POPC bilayer. The image rendering was done with VMD. Here peptides were shown in cartoon and their secondary structures were colored based on the default representation in VMD (α-helix: purple, 310-helix: blue, π-helix: red, extended-β: yellow, bridge-β: tan, turn: cyan, coil: white). Phospholipids were shown as thin gray lines, cholesterols as green sticks and GM1 as orange van der Waals spheres. (TIF) [file pone.0071308.s003.tif]

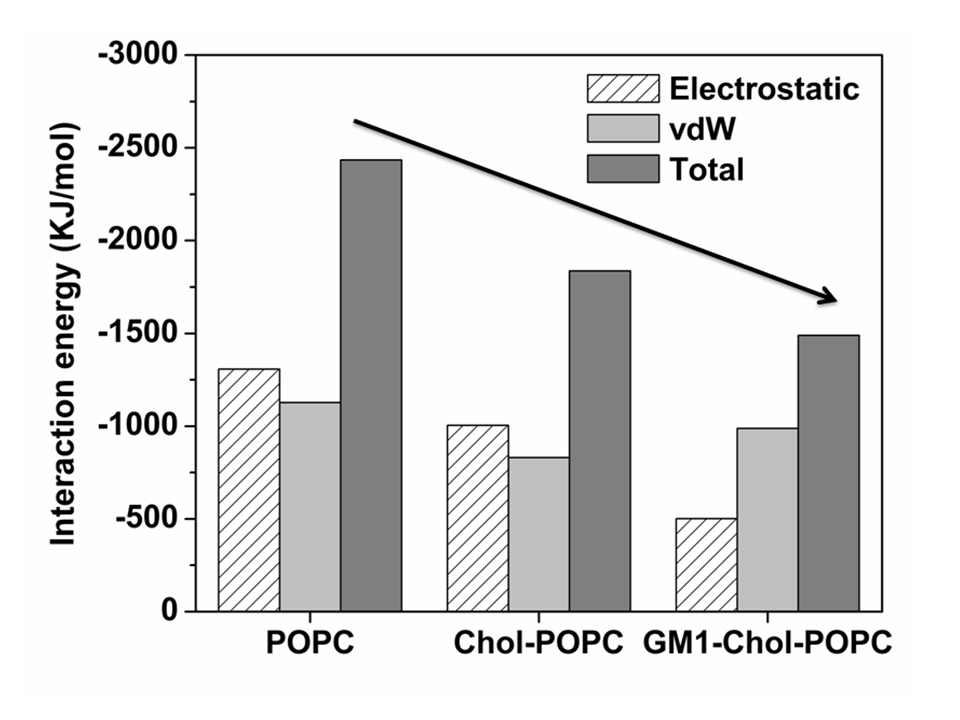

Supplement: Figure S4 — Lipid-peptide interaction energies in three different Aβ-monomer-membrane systems. The average values of last 100 ns data were presented here. (TIF) [file pone.0071308.s004.tif]

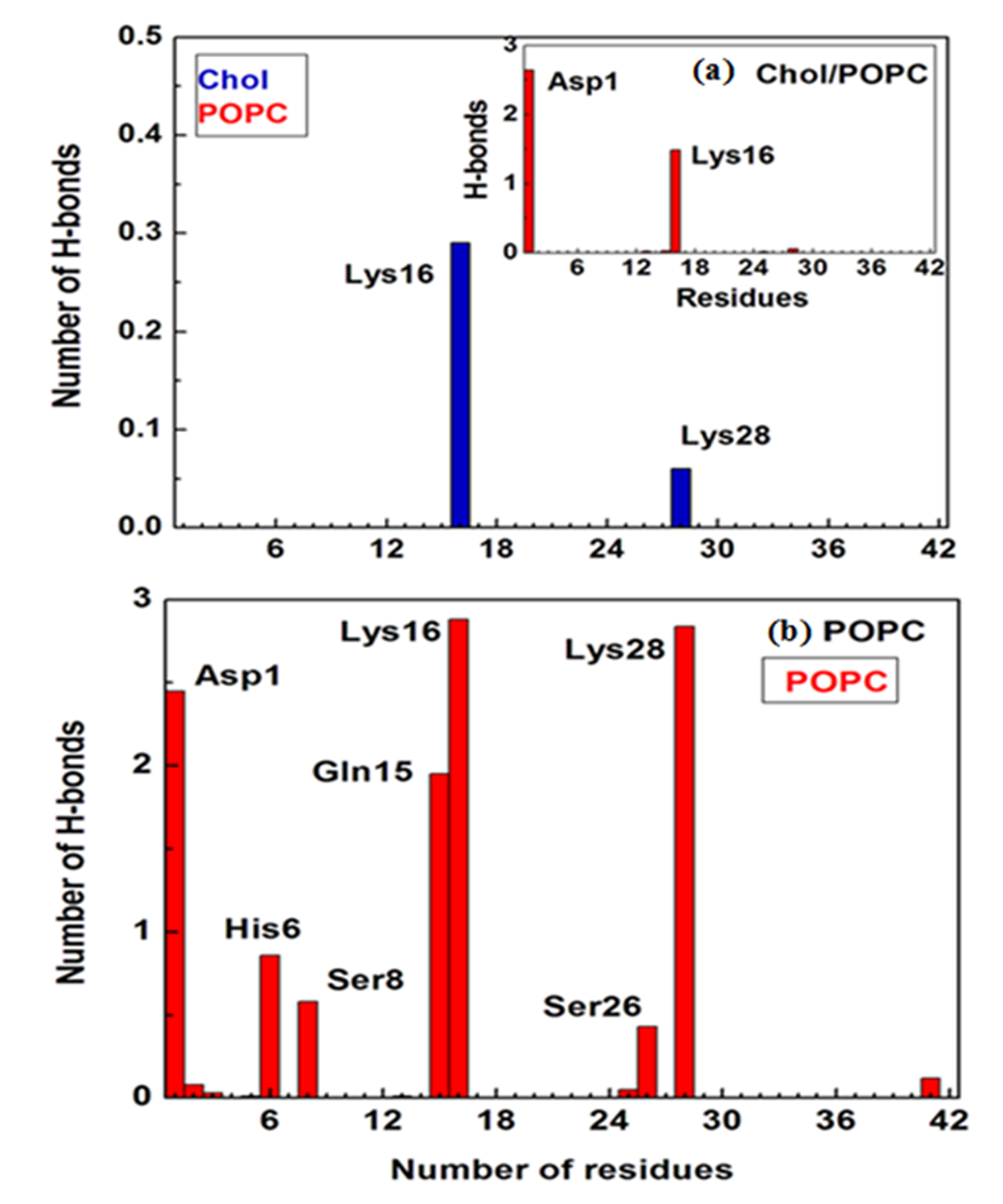

Supplement: Figure S5 — Number of Aβ-monomer-lipid H-bonds. H-bonds in (a) Chol/POPC and (b) POPC bilayers. (TIF) [file pone.0071308.s005.tif]

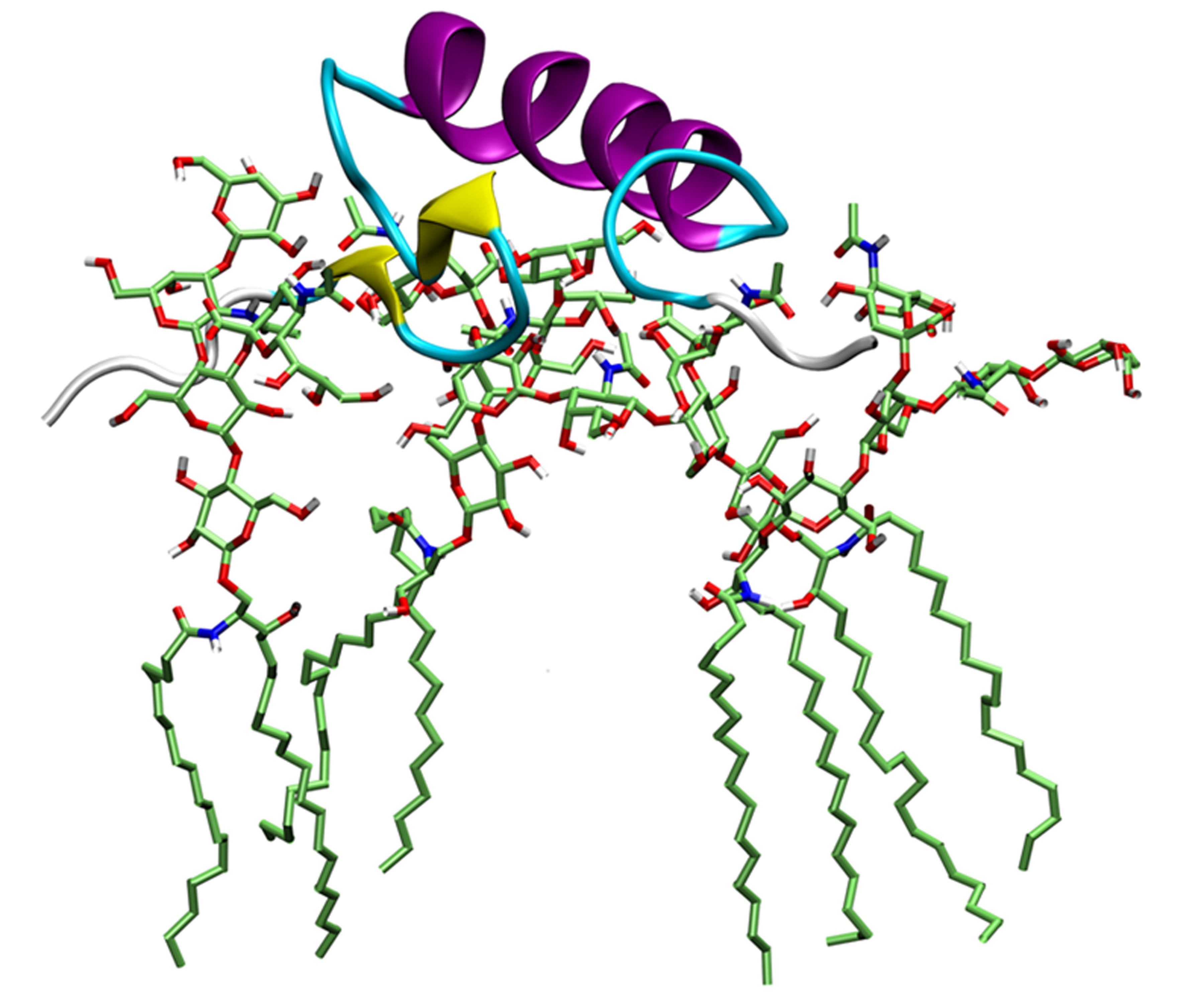

Supplement: Figure S6 — Aβ-monomer bound at the interface of four GM1 molecules. (TIF) [file pone.0071308.s006.tif]

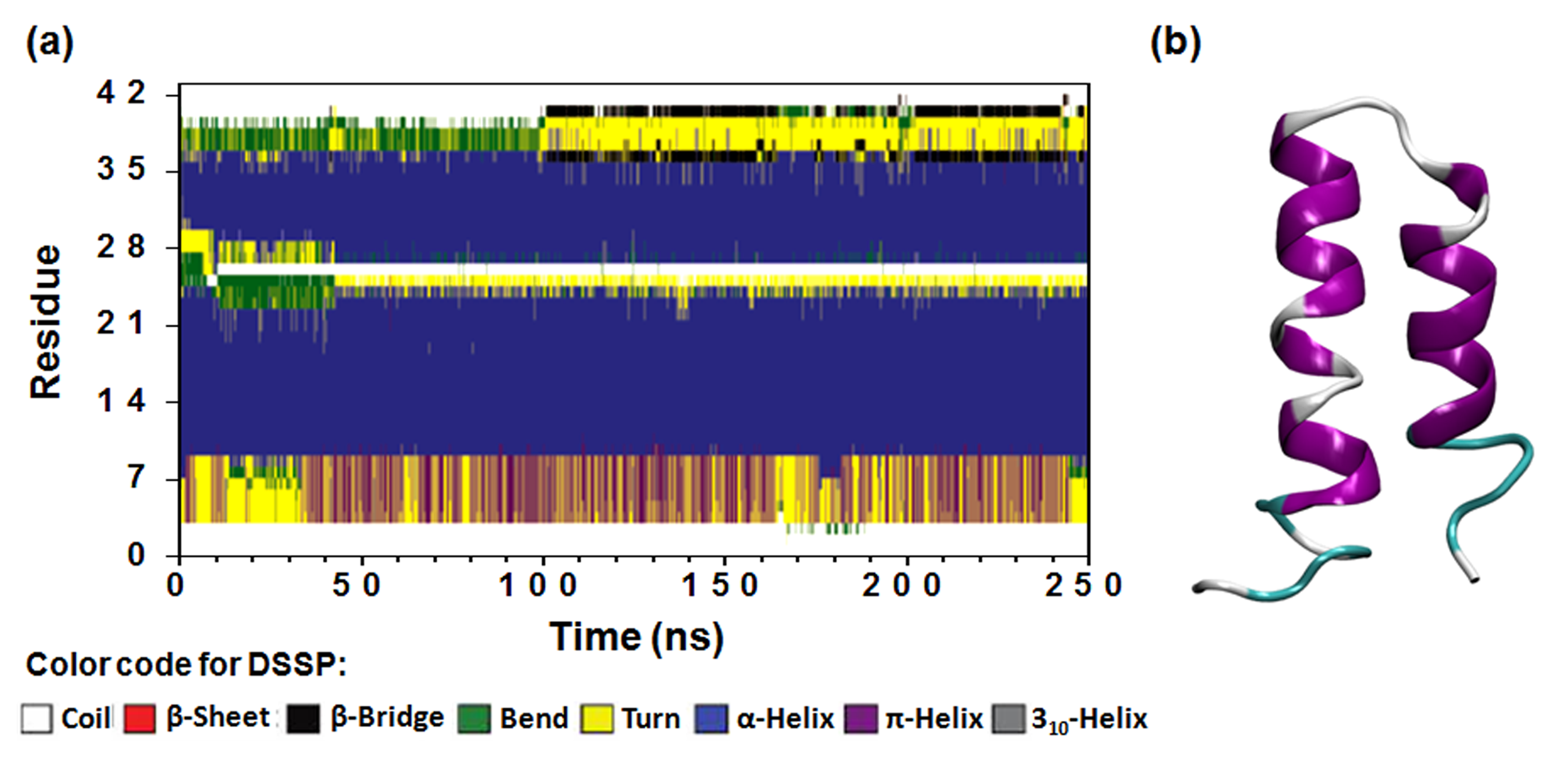

Supplement: Figure S7 — Secondary structure of Aβ-monomer in aqueous solution. (a) The secondary structure profile of Aβ in aqueous solution based on DSSP calculation. (b) The final snapshot of Aβ at 250 ns, prepared by VMD. The default color code of DSSP plot (a): random coil: white, β-sheet: red, β-bridge: black, bend: green, turn: yellow, α-helix: blue, π-helix: purple, 310-helix: gray. In snapshot (b) the secondary structures were colored based on the default representation in VMD (α-helix: purple, 310-helix: blue, π-helix: red, extended-β: yellow, bridge-β: tan, turn: cyan, coil: white). (TIF) [file pone.0071308.s007.tif]

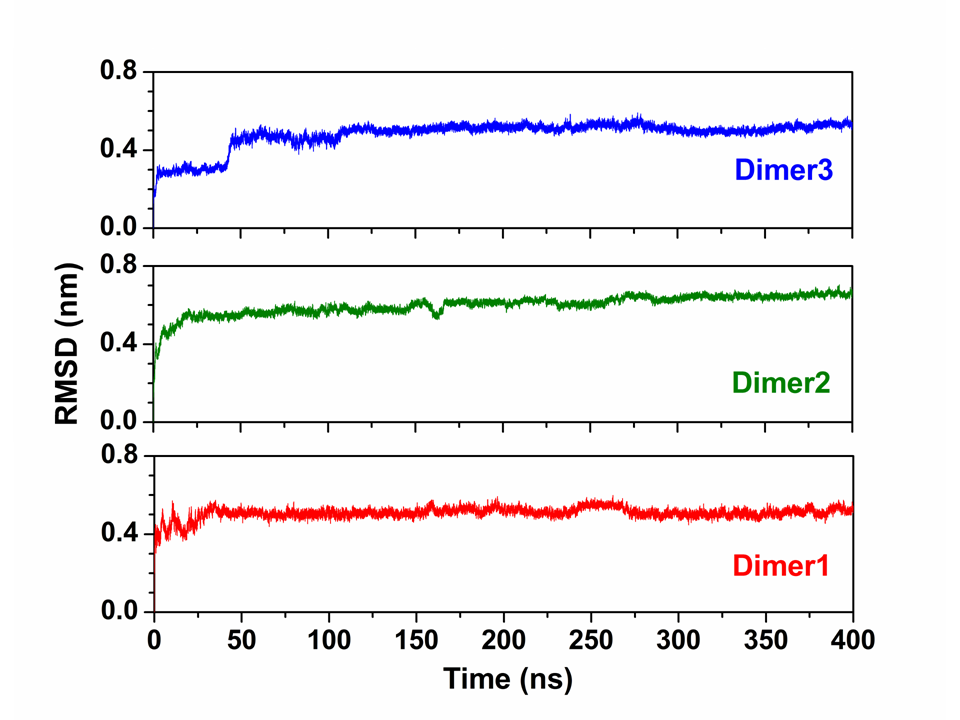

Supplement: Figure S8 — Root mean square deviations (RMSD) plotted against time for all three dimers. (TIF) [file pone.0071308.s008.tif]

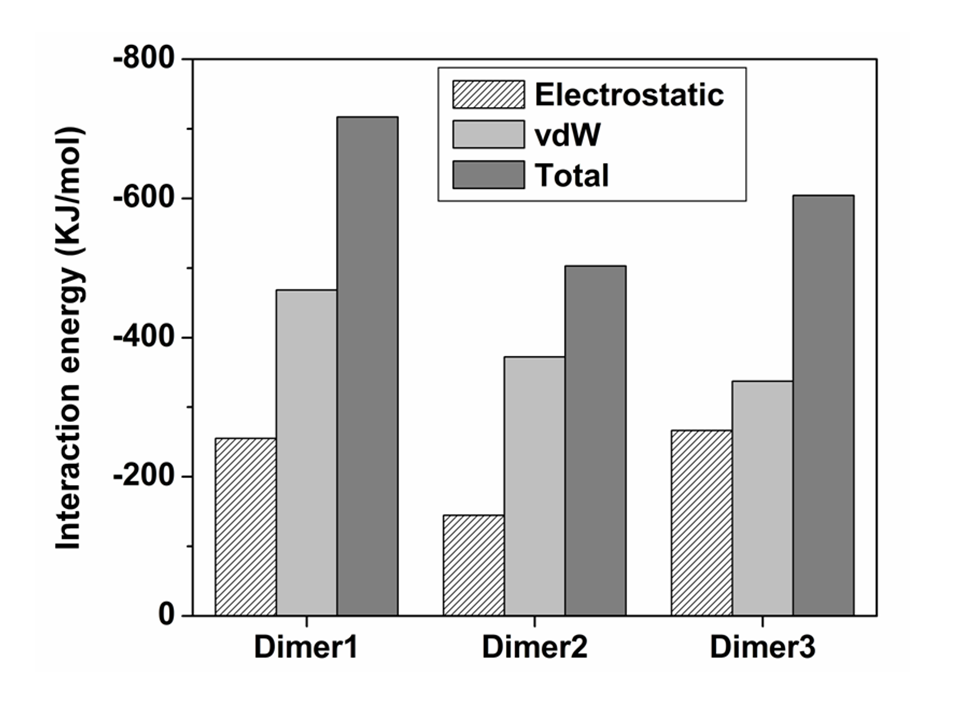

Supplement: Figure S9 — Aβ-Aβ interaction energies in dimers (last 200 ns average). (TIF) [file pone.0071308.s009.tif]

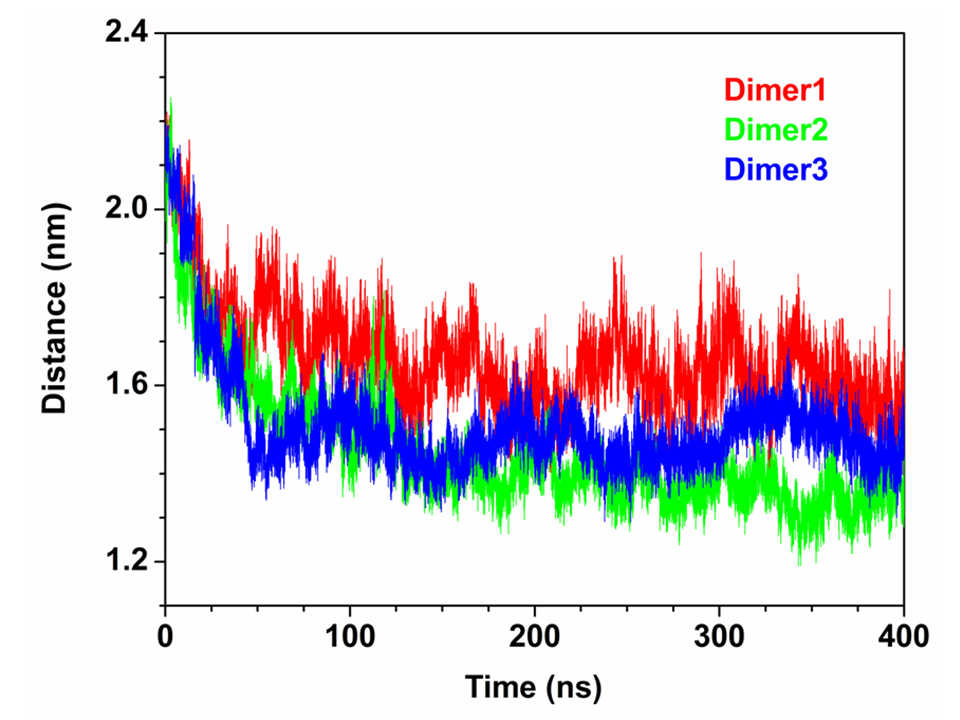

Supplement: Figure S10 — Preferential location of Aβ-dimers on GM1-contating membrane surface. Time dependence of the distance between the center of mass of Aβ-dimers and the average plane of phosphorus atoms in the contact monolayer. (TIF) [file pone.0071308.s010.tif]

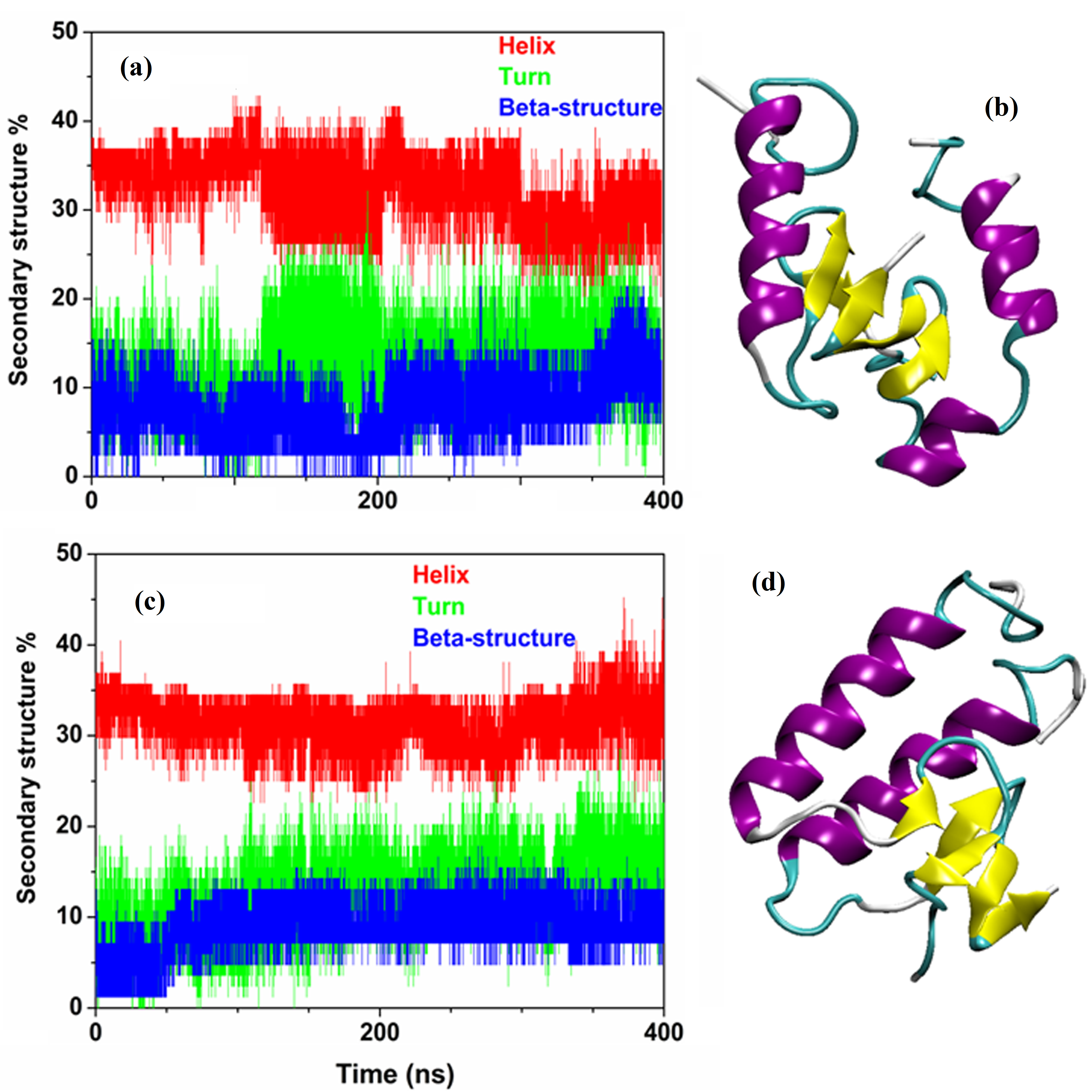

Supplement: Figure S11 — Secondary structures of Aβ-dimers. Time profile of secondary structural contents of (a) Dimer2 and (c) Dimer3 and their snapshots: (b) Dimer2 and (d) Dimer3 near the end of simulation. In snapshots the secondary structures of Aβ were colored based on the default representation in VMD (α-helix: purple, 310-helix: blue, π-helix: red, extended-β: yellow, bridge-β: tan, turn: cyan, coil: white). (TIF) [file pone.0071308.s011.tif]

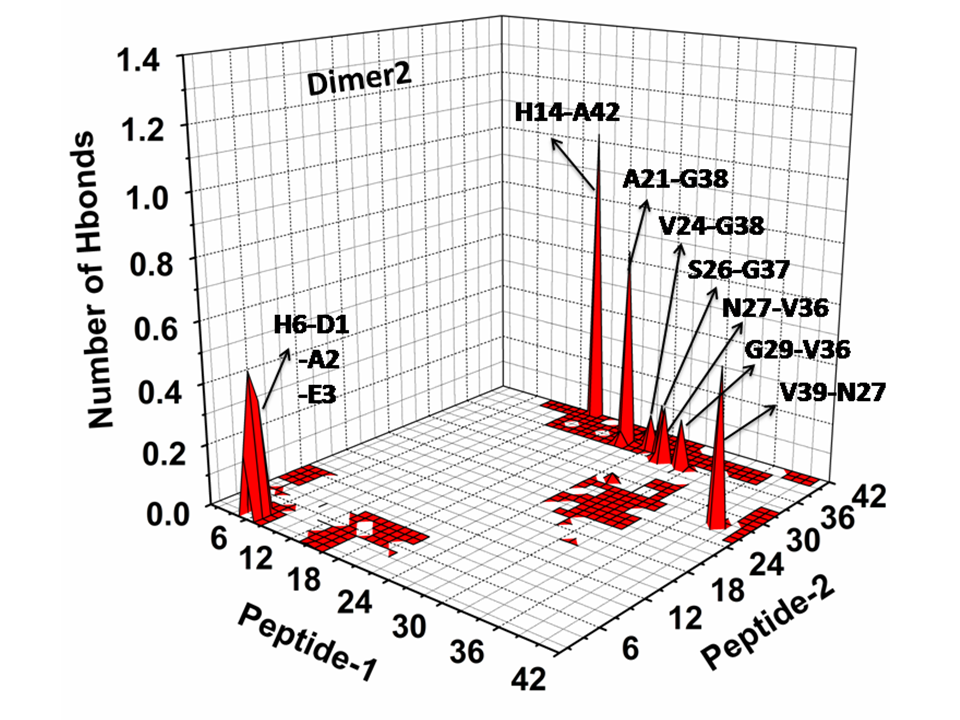

Supplement: Figure S12 — Inter-peptide H-bonds in Dimer2. 3D-plot showing the inter-peptide hydrogen-bonding interactions within Dimer2 (last 200 ns average). The notation used here for leveling H-bond between a pair of residues was XA-YB, where X was the amino acid residue of peptide-1 with its corresponding residue number A and Y was the residue of peptide-2 with its corresponding residue number B. (TIF) [file pone.0071308.s012.tif]

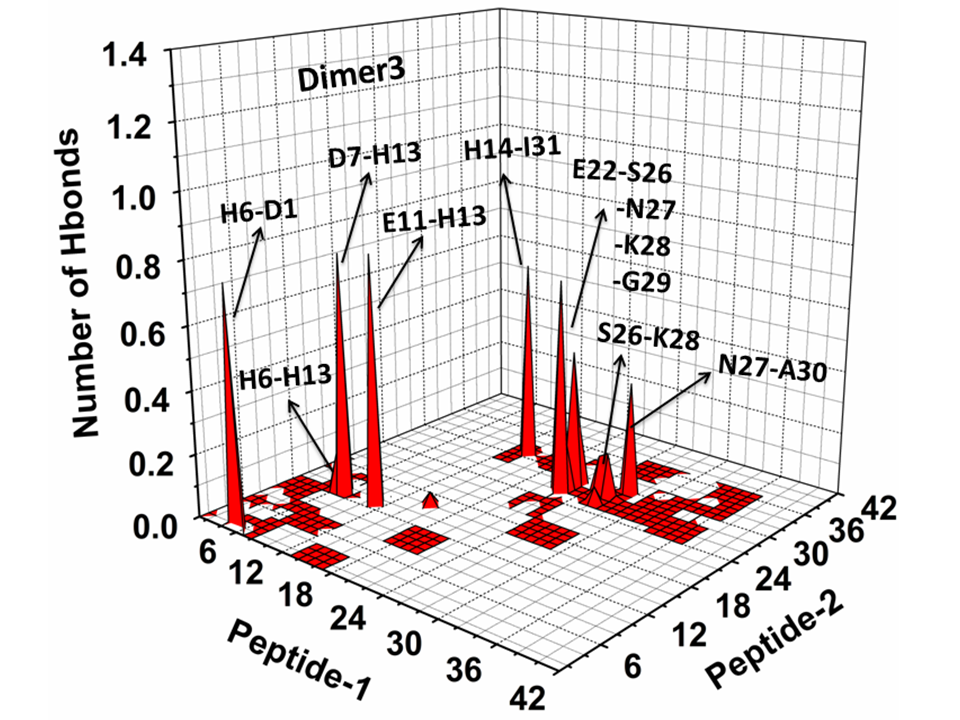

Supplement: Figure S13 — Inter-peptide H-bonds in Dimer3. 3D-plot showing the inter-peptide hydrogen-bonding interactions within Dimer3 (last 200 ns average). The notation used was same as Figure S12. (TIF) [file pone.0071308.s013.tif]
