# Supplementary material for: Binding, Conformational Transition and Dimerization of Amyloid-β Peptide on GM1-Containing Ternary Membrane: Insights from Molecular Dynamics Simulation
Source: PLoS One. 2013 Aug 9;8(8):e71308. doi: 10.1371/journal.pone.0071308 (PMC3739818; doi:10.1371/journal.pone.0071308)
Supplement: Table S1 — Properties of equilibrated bilayers. (DOC) [file pone.0071308.s014.doc]

| **Lipid bilayers** | **Order parameter of saturated tail** | **Order parameter of unsaturated tail** | **Area compressibility modulus (dyn/cm)** |
| --- | --- | --- | --- |
| **POPC** | 0.19 ± 0.04 | 0.17 ± 0.04 | 311.71 ± 62.9 |
| **Chol/POPC** | 0.26 ± 0.05 | 0.23 ± 0.05 | 1095.66 ± 41.87 |
| **GM1/Chol/POPC** | 0.31 ± 0.05 | 0.27 ± 0.05 | 1682.06 ± 37.24 |
