# Supplementary material for: Binding, Conformational Transition and Dimerization of Amyloid-β Peptide on GM1-Containing Ternary Membrane: Insights from Molecular Dynamics Simulation
Source: PLoS One. 2013 Aug 9;8(8):e71308. doi: 10.1371/journal.pone.0071308 (PMC3739818; doi:10.1371/journal.pone.0071308)
Supplement: Table S2 — Details of crucial Aβ-monomer-GM1 hydrogen bonding interactions. (DOC) [file pone.0071308.s015.doc]

| **Involvement of amino acid residues** | **Involvement of sugar moiety** |
| --- | --- |
| SER8: side chain OH and backbone CO | -NH-CO-CH_3_ and –OH groups of Neu5Ac |
| HIS13: ring NH | -COO^-^ and glycerol –OH groups of Neu5Ac |
| LEU34: backbone NH and CO | -NH-CO-CH_3_ group of Neu5Ac |
| VAL40: backbone NH and CO | -NH-CO-CH_3_ group of GalNAc, -OH and -CH_2_OH groups of Neu5Ac |
