# Supplementary material for: Binding, Conformational Transition and Dimerization of Amyloid-β Peptide on GM1-Containing Ternary Membrane: Insights from Molecular Dynamics Simulation
Source: PLoS One. 2013 Aug 9;8(8):e71308. doi: 10.1371/journal.pone.0071308 (PMC3739818; doi:10.1371/journal.pone.0071308)
Supplement: Table S3 — Properties of dimers (averaged over last 200 ns trajectory) on GM1-containing membrane. (DOC) [file pone.0071308.s016.doc]

| **Systems** | **Inter-peptide distance (nm)** | **Number of peptide-peptide contacts** | **Number of peptide- peptide H-bonds** |
| --- | --- | --- | --- |
| Dimer1 | 1.25 ± 0.05 | 347.81 ± 36.93 | 5.38 ± 1.75 |
| Dimer2 | 1.29 ± 0.04 | 229.54 ± 27.45 | 4.06 ± 1.68 |
| Dimer3 | 1.02 ± 0.04 | 224.67 ± 33.84 | 5.22 ± 1.58 |
