# Supplementary material for: Binding, Conformational Transition and Dimerization of Amyloid-β Peptide on GM1-Containing Ternary Membrane: Insights from Molecular Dynamics Simulation
Source: PLoS One. 2013 Aug 9;8(8):e71308. doi: 10.1371/journal.pone.0071308 (PMC3739818; doi:10.1371/journal.pone.0071308)
Supplement: Table S4 — Interaction of dimers with membrane surface (averaged over last 200 ns trajectory). (DOC) [file pone.0071308.s017.doc]

| **Systems** | **Aβ-membrane interaction energies (KJ/mol)** | **Number of Aβ-membrane contacts** | **Number of Aβ-membrane H-bonds** |
| --- | --- | --- | --- |
| Dimer1 | -1342.36 ± 84.79 | 968.68 ± 66.24 | 7.36 ± 2.13 |
| Dimer2 | -1811.00 ± 67.45 | 1197.98 ± 57.65 | 12.32 ± 2.19 |
| Dimer3 | -1361.84 ± 90.65 | 968.31 ± 69.79 | 5.85 ± 1.89 |
