# Supplementary material for: Binding, Conformational Transition and Dimerization of Amyloid-β Peptide on GM1-Containing Ternary Membrane: Insights from Molecular Dynamics Simulation
Source: PLoS One. 2013 Aug 9;8(8):e71308. doi: 10.1371/journal.pone.0071308 (PMC3739818; doi:10.1371/journal.pone.0071308)
Supplement: Table S5 — The formation time percentage (last 200 ns average) of intra- and inter-molecular salt-bridges in Aβ-dimers. Listed were those, which had more than 10% existence. (DOC) [file pone.0071308.s018.doc]

| **Dimer1** | | | **Dimer2** | | | **Dimer3** | | |
| --- | --- | --- | --- | --- | --- | --- | --- | --- |
| (+) ve charged residues | (-) ve charged residues | Probability of salt bridge formation (%) | (+) ve charged residues | (-) ve charged residues | Probability of salt bridge formation (%) | (+) ve charged residues | (-) ve charged residues | Probability of salt bridge formation (%) |
| ^1^K_28_^a^ | ^1^E_22_ | 18.37 | ^1^R_5_ | ^1^E_3_ | 40.46 | ^1^R_5_ | ^1^E_3_ | 24.26 |
| ^1^K_28_ | ^1^D_23_ | 34.11 | ^2^R_5_ | ^1^E_3_ | 28.00 | ^1^R_5_ | ^2^D_1_ | 45.44 |
| ^1^K_28_ | ^2^E_11_ | 10.27 | ^2^R_5_ | ^1^D_7_ | 31.24 | ^2^R_5_ | ^2^E_3_ | 72.34 |
| ^2^R_5_ | ^2^E_3_ | 52.55 | ^2^R_5_ | ^2^E_3_ | 71.73 | ^2^K_16_ | ^1^D_7_ | 18.33 |
| ^2^K_28_ | ^1^E_3_ | 18.35 | ^2^R_5_ | ^2^D_7_ | 21.18 | ^2^K_28_ | ^1^E_22_ | 45.75 |

**^a^**The prefixs denote peptide numbers (i.e., 1 for monomer-1 and 2 for monomer-2) within dimers, while sufixs indicate the residue numbers.
