# Supplementary material for: Binding, Conformational Transition and Dimerization of Amyloid-β Peptide on GM1-Containing Ternary Membrane: Insights from Molecular Dynamics Simulation
Source: PLoS One. 2013 Aug 9;8(8):e71308. doi: 10.1371/journal.pone.0071308 (PMC3739818; doi:10.1371/journal.pone.0071308)
Supplement: Table S6 — Details of inter-peptide hydrogen-bonding interactions within Dimer1. Listed were those which have H-bond ≥0.1. (DOC) [file pone.0071308.s019.doc]

| **Serial number** | **Residues involved** | **Type of interaction (residue1 – residue2)** |
| --- | --- | --- |
| 1 | 1Phe4-2Asn27 | Backbone - side chain |
| 2 | 1Arg5-2Val39 | Side chain, backbone - backbone |
| 3 | 1His6-2Ser26 | Backbone - side chain |
| 4 | 1Asp7-2Ser26 | Side chain - side chain |
| 5 | 1Tyr10-2Asn27 | Side chain - side chain |
| 6 | 1His13-2Ala42 | Side chain – C-terminus |
| 7 | 1Gly25-2His14 | Backbone - side chain |
| 8 | 1Ser26-2Tyr10 | Side chain, backbone - side chain |
| 9 | 1Ser26-2Glu11 | Side chain - side chain |
| 10 | 1Asn27-2Tyr10 | Side chain, backbone - side chain |
| 11 | 1Leu34-2Ile41 | Backbone - backbone |
| 12 | 1Val40-2Tyr10 | Backbone - side chain |
| 13 | 1Ile41-2His13 | Backbone - side chain |
| 14 | 1Ala42-2His13 | C-terminus - side chain |
