# Supplementary material for: Binding, Conformational Transition and Dimerization of Amyloid-β Peptide on GM1-Containing Ternary Membrane: Insights from Molecular Dynamics Simulation
Source: PLoS One. 2013 Aug 9;8(8):e71308. doi: 10.1371/journal.pone.0071308 (PMC3739818; doi:10.1371/journal.pone.0071308)
Supplement: Table S7 — Details of inter-peptide hydrogen-bonding interactions within Dimer2. Listed were those which have H-bond ≥0.1. (DOC) [file pone.0071308.s020.doc]

| **Serial number** | **Residues involved** | **Type of interaction (residue1 – residue2)** |
| --- | --- | --- |
| 1 | 1His6-2Asp1 | Side chain, backbone - side chain, backbone, N-terminus |
| 2 | 1His6-2Ala2 | Side chain - backbone |
| 3 | 1His6-2Glu3 | Side chain - backbone |
| 4 | 1His14-2Ala42 | Side chain - C-terminus |
| 5 | 1Ala21-2Gly38 | Backbone - backbone |
| 6 | 1Val24-2Gly38 | Backbone - backbone |
| 7 | 1Ser26-2Gly37 | Side chain, backbone - backbone |
| 8 | 1Asn27-2Val36 | Backbone - backbone |
| 9 | 1Gly29-2Val36 | Backbone - backbone |
| 10 | 1Val39-2Asn27 | Backbone - backbone |
