# Supplementary material for: Binding, Conformational Transition and Dimerization of Amyloid-β Peptide on GM1-Containing Ternary Membrane: Insights from Molecular Dynamics Simulation
Source: PLoS One. 2013 Aug 9;8(8):e71308. doi: 10.1371/journal.pone.0071308 (PMC3739818; doi:10.1371/journal.pone.0071308)
Supplement: Table S9 — Solvent-accessible surface area (SASA) for monomer (M) and dimers (D). (DOC) [file pone.0071308.s022.doc]

|  | **SASA (nm^2^)** | | | **Δ SASA (nm^2^) = D - (M + M)** | | |
| --- | --- | --- | --- | --- | --- | --- |
| **Systems** | **Hydrophobic** | **Hydrophilic** | **Total** | **Hydrophobic** | **Hydrophilic** | **Total** |
| **Monomer^a^** | 21.52 ± 0.50 | 13.64 ± 0.46 | 35.16 ± 0.65 | - | - | - |
| **Dimer1** | 33.55 ± 0.82 | 19.93 ± 0.70 | 53.47 ± 1.13 | (-) 9.49 | (-) 7.35 | (-) 16.85 |
| **Dimer2** | 33.24 ± 0.74 | 22.15 ± 0.75 | 55.39 ± 1.14 | (-) 9.8 | (-) 5.13 | (-) 14.93 |
| **Dimer3** | 33.59 ± 0.73 | 21.34 ± 0.62 | 54.93 ± 0.93 | (-) 9.45 | (-) 5.94 | (-) 15.39 |

**^a^**The data obtained from the simulation of Aβ-monomer bound to GM1/Chol/POPC bilayer
