# Supplementary material for: Binding, Conformational Transition and Dimerization of Amyloid-β Peptide on GM1-Containing Ternary Membrane: Insights from Molecular Dynamics Simulation
Source: PLoS One. 2013 Aug 9;8(8):e71308. doi: 10.1371/journal.pone.0071308 (PMC3739818; doi:10.1371/journal.pone.0071308)
Supplement: Text S1 — Description of additional data for Table S1. (DOC) [file pone.0071308.s023.doc]

**Supporting Information: Text S1.**

**Order parameter:** Molecular order parameter, S_mol_ [1], of POPC acyl tails were calculated using the following equation:

S_mol_=½<3cos^2^θ_n_-1>…………… [1]

Where, θ_n_ was the instantaneous angle between the n^th^ segmental vector, i.e., the (C_n-1_, C_n+1_) vector connecting (n-1) and (n+1) carbon atoms in lipid hydrocarbon chain and the bilayer normal (Z-axis). The angular brackets “<>” denote average over time and ensemble.

**Area compressibility modulus (K_area_)** gives the measure of mechanical strength of the membrane and was calculated using the following equation^2^


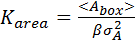
………………...[2]

Where, σ_A_ was the root mean square fluctuation in simulation box area. Consistent with our finding cholesterol was previously shown to increase the area compressibility modulus of membrane [2-3] and a very high
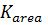
value (~1700 dyn/cm) was reported for sphingomyelin/Chol containing membrane [3]. The
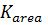
value for our sterol-free POPC bilayer was reasonably close to the experimental value of 278 dyn/cm for pure POPC membrane [4].

**References**

# Manna M, Mukhopadhyay C (2011) Molecular dynamics simulations of the interactions of kinin peptides with an anionic POPG bilayer. **Langmuir 27: 3713–3722.**

# Olsen BN, Schlesinger PH, Baker NA (2009) Perturbations of membrane structure by cholesterol and cholesterol derivatives are determined by sterol orientation. J Am Chem Soc 131: 4854–4865.

# Needham D, Nunn RS. (1990) Elastic deformation and failure of lipid bilayer membranes containing cholesterol. Biophys J 58: 997–1009.

1. Mathai JC, Tristram-Nagle S, Nagle JF, Zeidel ML (2007) Structural determinants of water permeability through the lipid membrane. J General Physiology 131:69–76.
