# Supplementary material for: Binding, Conformational Transition and Dimerization of Amyloid-β Peptide on GM1-Containing Ternary Membrane: Insights from Molecular Dynamics Simulation
Source: PLoS One. 2013 Aug 9;8(8):e71308. doi: 10.1371/journal.pone.0071308 (PMC3739818; doi:10.1371/journal.pone.0071308)
Supplement: Text S2 — Description of additional data for Table S3 and Figure S9. (DOC) [file pone.0071308.s024.doc]

**Supporting Information: Text S2.**

The data presented in Table S3 and Figure S9 shows that all dimers were stable. Though Dimer3 exhibited lowest peptide-peptide separation distance (that may be due to its stacking orientation), Dimer1 seemed to be the most stable one as it exhibited strongest Aβ-Aβ interaction (Figure S9), highest number of contact and hydrogen bonds (Table S3). The vdW interaction energies predominant (twice or more) over electrostatic contributions (Figure S9) - especially for Dimer1 and Dimer2, where the C-terminal hydrophobic segment of peptide was initially placed in adjacent position, while the N-terminal hydrophilic parts were away from each other (Figure S3a,c).
