# Supplementary material for: Binding, Conformational Transition and Dimerization of Amyloid-β Peptide on GM1-Containing Ternary Membrane: Insights from Molecular Dynamics Simulation
Source: PLoS One. 2013 Aug 9;8(8):e71308. doi: 10.1371/journal.pone.0071308 (PMC3739818; doi:10.1371/journal.pone.0071308)
Supplement: Text S3 — Description of additional data for Table S4 and Figure S10. (DOC) [file pone.0071308.s025.doc]

**Supporting Information: Text S3.**

**Preference of dimers to stay on the membrane surface**

The results (Figure S10 and Table S4) showed that all dimers exhibited preference to stay on the membrane surface. Interestingly, the order of dimer-lipid interactions: Dimer2>Dimer3>Dimer1 (Table S4) was exactly the reverse to the order of Aβ-Aβ interaction: Dimer2<Dimer3<Dimer1 (Figure S9) in dimers. These results indicated that, weaker binding to lipid favors stronger peptide-peptide interactions during dimerization.
